# Notch regulates vascular collagen IV basement membrane through modulation of lysyl hydroxylase 3 trafficking

**DOI:** 10.1007/s10456-021-09791-9

**Published:** 2021-05-06

**Authors:** Stephen J. Gross, Amelia M. Webb, Alek D. Peterlin, Jessica R. Durrant, Rachel J. Judson, Qanber Raza, Jan K. Kitajewski, Erich J. Kushner

**Affiliations:** 1 Department of Biological Sciences, University of Denver, Denver, CO 80210 USA; 2 HistoTox Labs, Boulder, CO USA; 3 Department of Physiology and Biophysics, University of Illinois, Chicago, IL USA

**Keywords:** Notch1, Angiogenesis, Blood vessels, Zebrafish, Development, Collagen IV, Rab10, Rab25, Lysyl hydroxlyase 3, Trafficking, Secretion, Small vessel disease

## Abstract

Collagen type IV (Col IV) is a basement membrane protein associated with early blood vessel morphogenesis and is essential for blood vessel stability. Defects in vascular Col IV deposition are the basis of heritable disorders, such as small vessel disease, marked by cerebral hemorrhage and drastically shorten lifespan. To date, little is known about how endothelial cells regulate the intracellular transport and selective secretion of Col IV in response to angiogenic cues, leaving a void in our understanding of this critical process. Our aim was to identify trafficking pathways that regulate Col IV deposition during angiogenic blood vessel development. We have identified the GTPase Rab10 as a major regulator of Col IV vesicular trafficking during vascular development using both in vitro imaging and biochemistry as well as in vivo models. Knockdown of Rab10 reduced de novo Col IV secretion in vivo and in vitro. Mechanistically, we determined that Rab10 is an indirect mediator of Col IV secretion, partnering with atypical Rab25 to deliver the enzyme lysyl hydroxylase 3 (LH3) to Col IV-containing vesicles staged for secretion. Loss of Rab10 or Rab25 results in depletion of LH3 from Col IV-containing vesicles and rapid lysosomal degradation of Col IV. Furthermore, we demonstrate that Rab10 is Notch responsive, indicating a novel connection between permissive Notch-based vessel maturation programs and vesicle trafficking. Our results illustrate both a new trafficking-based component in the regulated secretion of Col IV and how this vesicle trafficking program interfaces with Notch signaling to fine-tune basement membrane secretion during blood vessel development.

**Supplementary Information:**

The online version contains supplementary material available at 10.1007/s10456-021-09791-9.

## Introduction

Endothelial cells (ECs) are the cell type responsible for the bulk of embryonic blood vessel formation, eventually leading to an estimated 50,000 miles of vasculature by adulthood [1]. During development, new blood vessels emerge from pre-existing vasculature, a process termed angiogenesis [1–3]. During angiogenesis, ECs secrete a variety of proteins composing a planar protein network that encapsulates blood vessels, collectively termed basement membrane (BM). The bulk of the vascular BM is secreted during the angiogenic stages of development by ECs and later buttressed with mural cell interactions [4]. The BM not only provides a 50–200 nm thick static planar protein network on which ECs reside, but constitutes a dynamic and diverse extracellular environment vital to blood vessel integrity [5, 6]. The perivascular BM elements vary depending on anatomical location [7], but generally demonstrate an enrichment of macromolecular collagen IV (Col IV), laminins (4-1-1 and 5-1-1), perlecan, fibronectin and nidogen [4, 8] that are directly secreted by ECs and supportive cells [9–11]. For instance, laminins are anchored to Col IV by cross-linking of perlecan and nidogen, creating an exceptionally resilient co-polymer [12, 13]. In the absence of Col IV, BM integrity and cell integrin signaling are greatly diminished [14, 15]. In sprouting angiogenesis, ECs breakdown existing BM while simultaneously depositing it. How ECs orchestrate this feat, blending cell-autonomous signaling with tissue-level communication, is unclear and represents a void in our understanding of blood vessel development.

Blood vessels are exquisitely dependent on Col IV BM due to their inherent pressure demands as a fluid transport system. Disruption in Col IV bioavailability during blood vessel development is the basis of small vessel disease (SVD) in which Col IV point mutations promote intracellular retention or degradation of Col IV, limiting its perivascular deposition. This reduced Col IV secretion in SVD is associated with intracerebral hemorrhage, typically resulting in death or profound disability [16]. Genetic ablation of Col IV in mice does not prevent angiogenesis, per se, but is embryonically lethal due to an inability of blood vessels to resist the mechanical strain of circulation resulting in widespread hemorrhage [15]. Col IV itself is an obligate heterotrimer made of 3 alpha chains forming a long triple helix [17]. Trimer formation is, in part, achieved through lysyl hydroxylase (LH) 1-3 (genes Procollagen-Lysine, 2-Oxoglutarate 5-Dioxygenase (PLOD1,2,3)) that catalyze hydroxylysine formation, without which stable heterotrimer formation is abolished. LH1 and LH2 are restricted to the endoplasmic reticulum (ER). LH3 demonstrates an affinity for Col IV over other collagen subtypes, found both in the ER and on post-Golgi vesicles and is secreted [18]. Indeed, Col IV homeostasis is important for both blood vessel development and maintenance.

Akin to transcriptional networks, vesicular trafficking programs are complex and likely comprised unique organotypic signatures that are fundamental to tissue form and function. In terms of Col IV, how Col IV is transported, targeted to the basal membrane, interfaces with degradative organelles or intersects with other proteins/enzymes during angiogenesis is mostly unknown. It is understood that proangiogenic molecules such as vascular endothelial growth factor (VEGF) or fibroblast growth factor (FGF) are associated with secretion of ECM proteins during angiogenesis; however, it is not known how such angiocrine signaling affects BM trafficking. Furthermore, how permissive blood vessel maturation programs, such as Notch signaling [19], interface with BM trafficking is largely uncharacterized.

Here, we describe a novel level of Col IV regulation that leverages vesicular transport to precisely modulate Col IV secretion in ECs during blood vessel development. Specifically, we demonstrate that Rab10 and Rab25 GTPases govern the transport and delivery of LH3 to Col IV vesicles staged for secretion. In the absence or inactivation of Rab10 or Rab25, LH3 trafficking is halted and Col IV secretion is abolished. We also demonstrate a first of its kind connection between permissive Notch signaling and control of Rab10 GTPase activity trafficking to regulate Col IV bioavailability during blood vessel development. Our data indicates that Notch activation modulates Rab10-activiting guanine exchange factor DENNd4C to regulate intracellular transport of LH3 and downstream Col IV secretion. Overall, our results illustrate a novel Col IV trafficking network that controls Col IV BM deposition during sprouting angiogenesis.

## Results

### Loss of Rab10 impairs endothelial basement membrane secretion

**Fig. 1:**
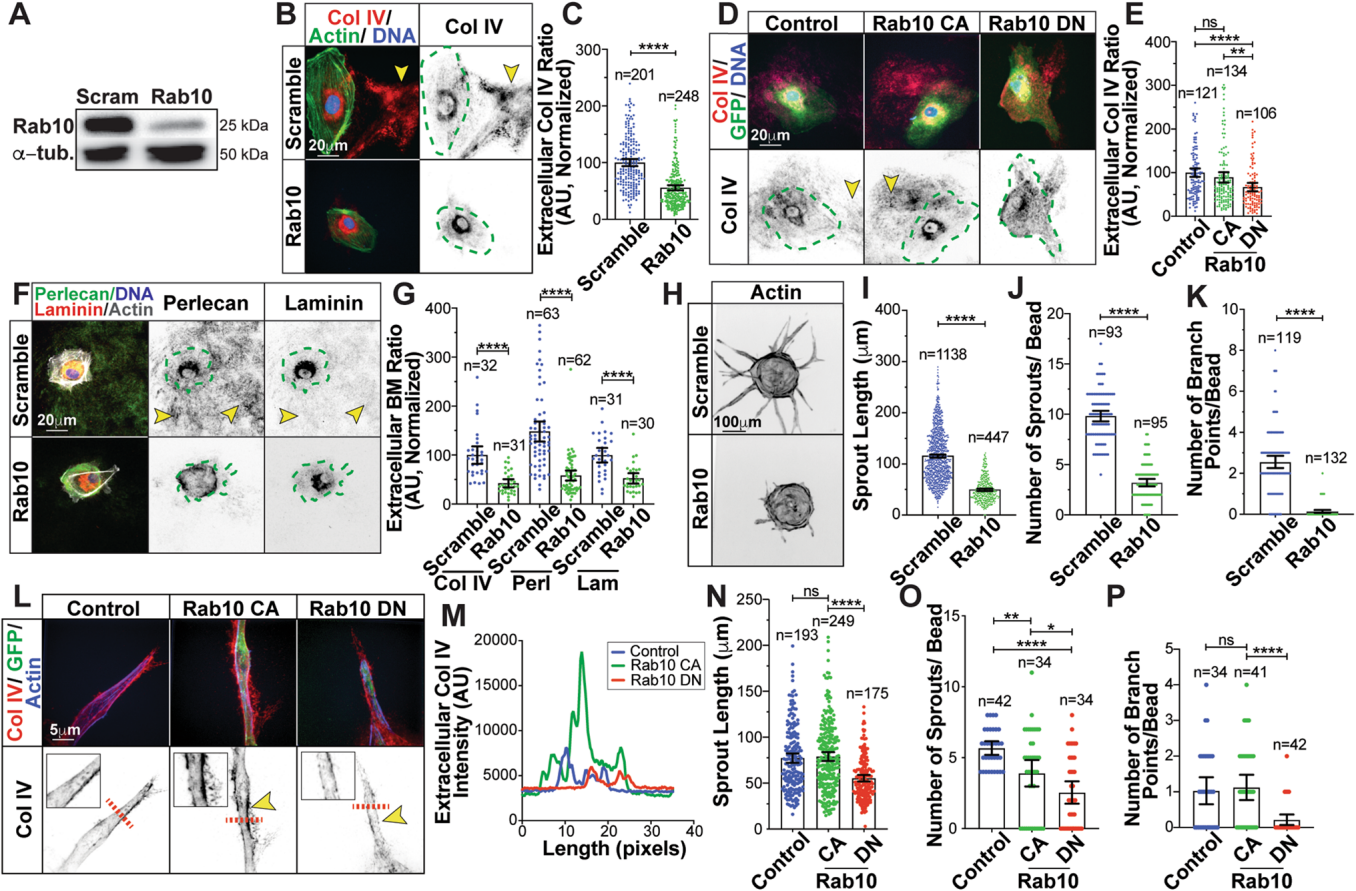
Loss of Rab10 impairs endothelial basement membrane secretion. (**a**) Immunoblot of Rab10 in ECs transfected with either scrambled or Rab10 siRNA and probed for indicated proteins. (**b**) Representative images of scramble or Rab10 siRNA-treated ECs and stained for collagen IV (Col IV) (red), actin (green), and DNA (blue). Dotted green line indicates cell outline. Arrowheads denote extracellular Col IV secretion. (**c**) Graph of Col IV extracellular ratio in scramble or Rab10 siRNA-treated ECs. *N* number of cells. (**d**) Representative image of ECs expressing GFP only (control), GFP-Rab10 constitutively active (CA) or GFP-Rab10 dominant negative (DN), stained for Col IV (red) and DNA (blue). Dotted green line indicates cell outline. Arrowheads denote extracellular Col IV secretion. (**e**) Graph of Col IV extracellular ratio in ECs expressing GFP only (control) or GFP-Rab10 CA/DN. *N* number of cells. (**f**) Representative images of scramble or Rab10 siRNA-treated ECs and stained for laminin (red) perlecan (green), actin (grey), and DNA (blue). Dotted green line indicates cell outline. Arrowheads denote extracellular Col IV secretion. (**g**) Graph of extracellular basement membrane (BM) ratio in scramble or Rab10 siRNA-treated ECs between indicated secreted proteins. *N* number of cells. (**h**) Representative image of fibrin-bead sprouts between indicated siRNA treatment groups. Sprouts were stained for actin (grey) to delineate sprout morphology. (**i**–**k**) Graphs of sprouting parameters for scramble or Rab10 siRNA-treated sprouts. *N* number of measurements. (**l**) Representative images of ECs expressing GFP only (control) or GFP-Rab10 CA/DN in fibrin-bead sprouts. Dotted red line indicates line scan of (**m**). Arrowheads denote extracellular Col IV secretion. (**m**) Line scan measurement of Col IV fluorescence intensity shown in (**l**). (**n**–**p**) Graph of sprouting parameters for GFP only (control) or GFP-Rab10 CA/DN expressing sprouts. *N*  number of measurements. For all experiments, data represented as mean ± 95% confidence intervals. Black bars indicate comparison groups with indicated *p*-values. All *p*-values are from two-tailed Student’s *t*-test from at least three experiments. *p ≤ 0.05; **p ≤ 0.01; ***p ≤ 0.001; ****p ≤ 0.0001; *ns* not significant

Based on previous literature implicating the GTPase Rab10 as a Col IV trafficking mediator [5], we first sought to determine if Rab10 was involved in Col IV secretion in primary ECs. Here we used single ECs plated on coverslips to be able to ascribe secretion characteristics to individual cells. ECs demonstrated a robust secretion of Col IV marked by long trails leading back to individual ECs. Rab10 knockdown significantly diminished Col IV secretion with a limited amount of Col IV being deposited under the ventral/basal surface of the EC (Fig. 1a–c). Next, we transduced ECs with constitutively active (CA, Q68L) or a dominant negative (DN, T23N) Rab10 fused to a green fluorescence protein (GFP) that demonstrated differential vesicle localization (Figure S1a) and was previously validated [20]. ECs expressing a GFP-Rab10 CA or control GFP did not show any difference in Col IV secretion compared with each other. However, expression of the GFP-Rab10 DN significantly reduced Col IV secretion compared to both WT and CA Rab10 (Fig. 1d, e). Secreted Col IV is typically associated with other BM proteins such as perlecan and laminins [21, 22]. Rab10 knockdown also significantly blunted the secretion of these vascular BM proteins (Fig. 1f, g). It is possible that reduced Col IV secretion could be related to diminish migratory capacity in ECs lacking Rab10. To factor this out, we performed a scratch wound assay as a gauge of cell motility. There was no effect of Rab10 knockdown on cell migration (Figure S1b, c). Additionally, we determined that Rab10 did not affect apoptotic tendency by measuring cleaved caspase-3 levels or proliferation (Figure S1d–f). These results suggest that Rab10 is associated with Col IV secretion in ECs.

To determine if Rab10 affected 3-dimensional (3D) sprouting behaviors we employed a fibrin-bead assay in which ECs form multicellular sprouts in a fibrin matrix [23]. Loss of Rab10 resulted in a 70–90% reduction in sprout parameters compared with controls (Fig. 1h–k). To further probe how loss and gain of function of Rab10 affected Col IV secretion in 3D sprouting, we mosaically transduced GFP-Rab10 DN and CA mutants into growing sprouts. Staining non-permeabilized sprouts for secreted Col IV showed that ECs expressing the DN form of Rab10 had lower levels of perivascular Col IV, while the CA Rab10 mutant showed qualitatively elevated Col IV secretion compared with a non-transduced control (Fig. 1l, m; S1g–I). In comparing sprout morphology, only the DN version of Rab10 impaired sprout length and number of branch points in reference to GFP and CA Rab10 expressing ECs (Fig. 1n–p). Interestingly, both Rab10 CA and DN impaired sprout formation, the DN variant to a greater magnitude, compared with WT Rab10 (Fig. 1o). Given the lack of extracellular Col IV we treated fibrin-bead sprouts with the pan secretion inhibitor brefeldin A (BFA) to determine if loss of Rab10 resembled a general secretion defect by comparing sprouting phenotypes. Like Rab10 knockdown, administration of BFA significantly diminished all sprouting parameters, indicating that Rab10 may also affect general secretion mechanisms (Figure S1j–m). Overall, these results indicate that Rab10 may be necessary for in vitro sprouting.

### Rab10 influences intracellular Col IV protein stability

**Fig. 2:**
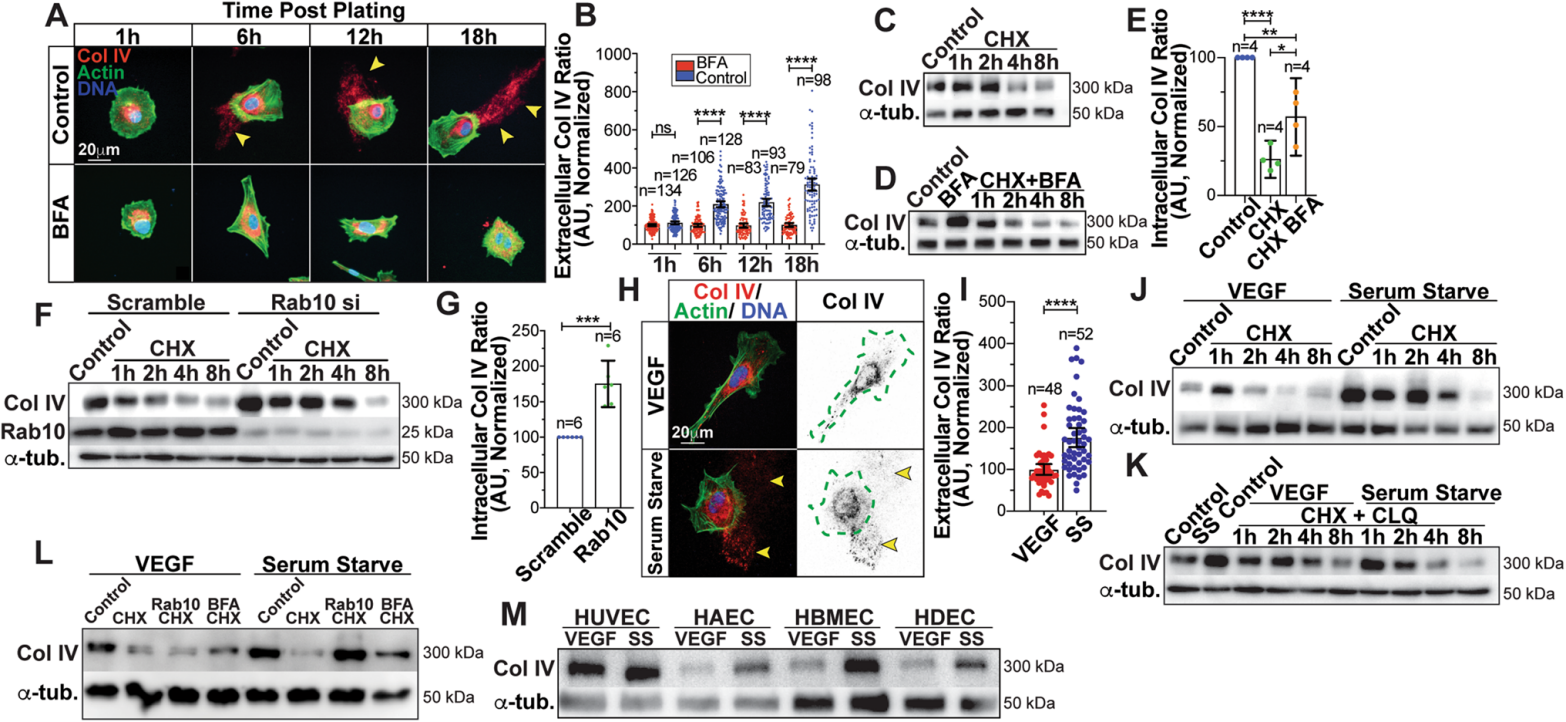
Collagen IV follows a post-Golgi secretory pathway. (**a**) Representative images of ECs treated with brefeldin A (BFA) at indicated time points after BFA exposure. Cells were stained for collagen IV (Col IV) (red), actin (green), and DNA (blue). Arrowheads denote extracellular Col IV secretion. (**b**) Graph of Col IV extracellular ratio in control or BFA-treated ECs at indicated time points after BFA exposure. *N* number of cells. (**c**) Immunoblot of Col IV in ECs treated with cycloheximide (CHX) (20 mg/ml) and probed for indicated proteins. (**d**) Immunoblot of Col IV in ECs treated with both CHX + BFA and probed for indicated proteins. (**e**) Graph of Col IV protein accumulation in ECs treated with CHX, and both CHX + BFA. Col IV accumulations were normalized to α-tubulin levels. *N* number of replicates. (**f**) Immunoblot of Col IV in ECs transfected with either scramble or Rab10 siRNA and treated with CHX. EC lysate probed for indicated proteins. (**g**) Graph of Col IV levels in ECs transfected with either scramble or Rab10 siRNA and treated with CHX. Col IV accumulations were normalized to α-tubulin levels. *N* number of replicates. (**h**) Representative images of ECs cultured in VEGF-containing or serum-starve (SS) media and stained for Col IV (red), actin (green), and DNA (blue). Dotted green line indicates cell outline. Arrowheads denote extracellular Col IV secretion. (**i**) Graph of Col IV extracellular ratio of ECs cultured in VEGF-containing or SS media. *N* number of cells. (**j**) Immunoblot of Col IV in ECs cultured in VEGF-containing or SS media treated with CHX for the indicated time and probed for indicated proteins. (**k**) Immunoblot of Col IV in ECs cultured in VEGF-containing or SS media treated with CHX and chloroquine (CLQ) (10 µM) for the indicated times and probed for indicated proteins. (**l**) Immunoblot of Col IV in ECs transfected with either scramble or Rab10 siRNA and cultured in VEGF-containing or SS media treated with CHX and/or BFA for 8hrs and probed for indicated proteins. (**m**) Immunoblot of Col IV in Human umbilical vein ECs (HUVEC), Human aortic ECs (HAEC), Human brain microvascular ECs (HBMEC) and Human dermal ECs (HDEC) cultured in either VEGF-containing or SS media and probed for indicated proteins. For all experiments, data represented as mean ± 95% confidence intervals. Black bars indicate comparison groups with indicated *p*-values. All *p*-values are from two-tailed Student’s *t*-test from at least three experiments. *p ≤ 0.05; **p ≤ 0.01; ***p ≤ 0.001; ****p ≤ 0.0001; *ns* not significant

Next, we sought to understand how Rab10 impacts Col IV secretion by first investigating normal Col IV cellular turnover in ECs. ECs were again incubated with the secretion-disrupting compound BFA. BFA treatment ablated Col IV secretion, suggesting that Col IV itself or its regulators use a classical post-Golgi trafficking route (Fig. 2a, b). Next, we inhibited new protein synthesis to determine the half-life of intracellular Col IV in the absence of secretion using cycloheximide (CHX) with and without BFA. Cycloheximide addition reduced the intracellular Col IV pool by ~ 75% at 4 h (Fig. 2c). Strikingly, addition of the secretion inhibitor BFA doubled the Col IV content in the cycloheximide condition (Fig. 2d, e), indicating that at least half of the Col IV decay was due to secretion. Knockdown of Rab10 closely mimicked BFA-induced intracellular Col IV retention (Fig. 2f, g). These results demonstrate the EC Col IV pool is rapidly secreted and knockdown of Rab10 resembles chemically induced inhibition of secretion, thus Rab10 may play a mechanistic role in this pathway.

Vascular endothelial growth factor (VEGF) is one of the most well-characterized proangiogenic factors essential for angiogenesis and is involved with regulation of migration, proliferation and blood vessel stabilization programs. [24–28]. Given VEGF’s involvement in antagonizing blood vessel stabilization programs that typically involve elevated basement deposition, we sought to determine how VEGF influenced Col IV secretion. Strikingly, VEGF supplementation significantly impeded Col IV secretion in freely migrating ECs compared with ECs in serum-starvation (SS) culture media (Fig. 2h, i). Moreover, VEGF supplementation showed a concentration-dependent reduction in Col IV, laminins and perlecan secretion (Figure S2a–d). Once again, we used cycloheximide to determine the half-life of intracellular Col IV with VEGF stimulation. In line with the general lack of secretion in the VEGF-treated ECs, VEGF stimulation increased the rate of the Col IV depletion compared with a SS control (Fig. 2j). Incubation with the lysosomal inhibitor chloroquine rescued VEGF-mediated loss of Col IV, indicating that VEGF-induced Col IV destruction via the lysosome (Fig. 2k). We next compared how Rab10 affected intracellular Col IV levels in VEGF-exposed and SS states. Knockdown of Rab10 in the presence of VEGF still resulted in Col IV degradation as compared with the SS state or BFA control when treated with cycloheximide (Fig. 2l). This finding promotes the notion that VEGF-induced Col IV degradation does not involve Rab10, suggesting that Rab10 is participating in a secretory, not a degradative pathway. To ensure this degradative response to VEGF was not restricted to human umbilical vein ECs, we assayed for intracellular Col IV levels in human aortic, human brain, human microvascular and human dermal primary ECs. Across all primary cell lines, Col IV levels were reduced in the VEGF-supplemented state compared with SS ECs (Fig. 2m), suggesting this is likely a global endothelial response.

### Rab10 and Rab25 work in combination to traffic LH3 to collagen IV-containing vesicles

**Fig. 3:**
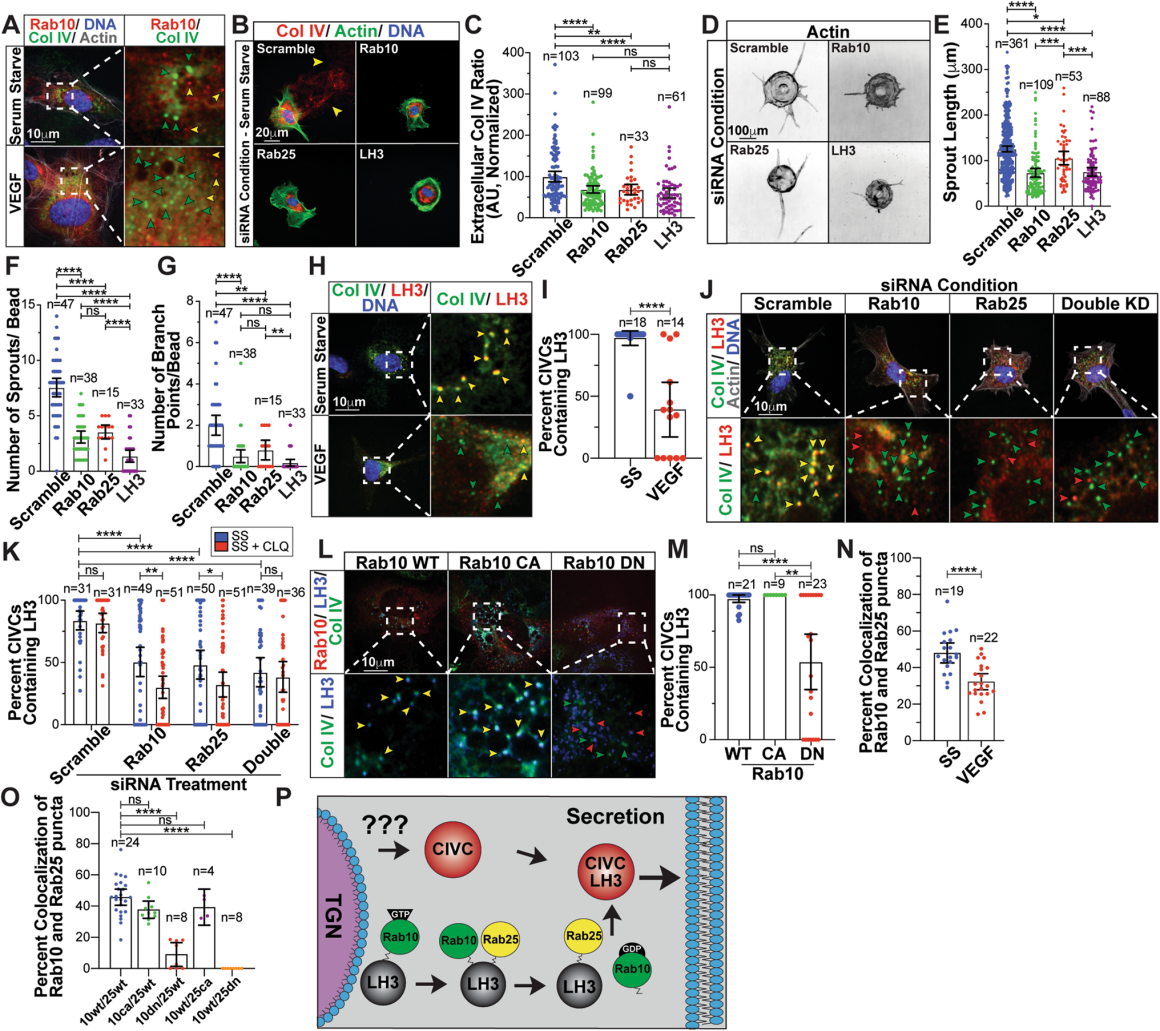
Rab10 and Rab25 work in combination to traffic LH3 to CIVC vesicles. (**a**) Representative images of ECs expressing RFP-Rab10 wild-type (WT) and stained for collagen IV (Col IV) (green). Green arrowheads indicate collagen IV-containing (CIVC) vesicles only and yellow arrowheads indicate Rab10 puncta only. (**b**) Representative images of scramble, Rab10, Rab25, or LH3 siRNA-treated ECs and stained for Col IV (red), actin (green), and DNA (blue). Arrowheads denote extracellular Col IV secretion. (**c**) Graph of extracellular Col IV ratio of scramble, Rab10, Rab25, or LH3 siRNA-treated ECs cultured in serum-starve (SS) media. (**d**) Representative images of fibrin-bead sprouts between indicated siRNA treatment groups. Sprouts were stained for actin (grey) to delineate sprout morphology. (**e**–**g**) Graphs of sprouting parameters for scramble, Rab10, Rab25, or LH3 siRNA-treated sprouts. (**h**) Representative images of ECs cultured in VEGF-containing or SS media and stained for Col IV (green), LH3 (red), and DNA (blue). Green arrowheads indicate CIVC vesicles only and yellow arrowheads indicate co-localized puncta. (**i**) Graph of percent CIVC vesicles co-localized with LH3 in ECs cultured in VEGF-containing or SS media. (**j**) Representative images of scramble, Rab10, Rab25, or both Rab10/25 siRNA-treated ECs and stained for Col IV (green), LH3 (red), actin (grey), and DNA (blue). Yellow arrowheads indicate co-localized puncta only, green arrowheads indicate Col IV only puncta, and red arrowheads indicate LH3 puncta only. (**k**) Graph of percent CIVC vesicles co-localized with LH3 in scramble, Rab10, Rab25, or LH3 siRNA-treated conditions cultured in SS media or SS media with CLQ (10 µM). (**l**) Representative images of ECs expressing RFP-Rab10 WT, RFP-Rab10 constitutively active (CA), or RFP-Rab10 dominant negative (DN), stained for Col IV (green) and LH3 (blue). Yellow arrowheads indicate co-localized puncta only, green arrowheads indicate Col IV puncta, and red arrowheads indicate LH3 puncta. (**m**) Graph of percent CIVC vesicles co-localized with LH3 in ECs expressing RFP-Rab10 WT/CA/DN. (**n**) Graph of percent Rab10 wild-type (WT) puncta co-localized with Rab25 in either VEGF-containing or SS media. (**o**) Graph of percent Rab10 puncta co-localized with Rab25 in ECs transfected with indicated constructs. (**p**) Schematic diagram showing how Rab10 and Rab25 function to coordinate delivery of LH3 to CIVC vesicles for proper secretion of Col IV. For all graphs *n* number of cells unless otherwise indicated. For all experiments, data represented as mean ± 95% confidence intervals. Black bars indicate comparison groups with indicated *p*-values. All *p*-values are from two-tailed Student’s *t*-test from at least three experiments. *p ≤ 0.05; **p ≤ 0.01; ***p ≤ 0.001; ****p ≤ 0.0001; *ns* not significant

Given the strong effect of Rab10 on both Col IV secretion and bioavailability, we originally hypothesized that Rab10 was directly mediating Col IV vesicular trafficking (e.g., physically attached to Col IV vesicles). However, we did not observe Rab10 co-localization with Col IV-containing (CIVC) vesicles (Fig. 3a), these vesicles are relatively large, electron dense and shown to house Col IV staged for secretion [29, 30]. This lack of co-localization strengthened the hypothesis that Rab10 may play an indirect role in Col IV trafficking. To this end, the lysyl hydroxylase 3 (LH3) enzyme has been shown to be critical for Col IV post-Golgi protein stability and secretion [29]. Additionally, it has been previously reported that LH3 trafficking required both Rab10 and Rab25 [30]. To determine if Rab10 was involved in LH3 trafficking, we first compared Col IV secretion between Rab10 and Rab25 knockdowns to LH3 knockdowns. Loss of Rab10 or Rab25 phenocopied the reduced EC Col IV secretion observed with LH3 KD (Fig. 3b, c), indicating that both Rab10 and Rab25 impact Col IV secretion to a similar magnitude compared with LH3 depletion. Rab10 and Rab25 knockdown also significantly reduced sprouting parameters similar to LH3 knockdown in reference to a control group (Fig. 3d–g). These data demonstrate that Rab10 and Rab25 equally affect Col IV secretion and sprouting parameters in comparison to LH3 knockdown.

Reasoning that LH3 is the cargo of Rab10 and Rab25, we first determined the efficiency of LH3 transport to CIVC vesicles in the VEGF-supplemented and SS state in which Col IV secretion is greatly affected. We observed that in the SS state, LH3 co-localized with CIVC vesicles 99% of the time, while VEGF supplementation reduced LH3/CIVC vesicle co-localization by ~ 50% (Fig. 3h, i). Given the SS culture condition produced a near perfect co-localization between LH3 and CIVC vesicles, we used this SS condition to test how loss of Rab10 and Rab25 trafficking impacts LH3 transport to CIVC vesicles. Knockdown of Rab10, Rab25 or combination significantly reduced LH3 and CIVC vesicle co-localization compared with controls (Fig. 3j, k). In this experiment, chloroquine was added to prevent both LH3 and Col IV degradation to determine what fraction may be lysosomally degraded when Rab10 and Rab25 trafficking mediators are absent. Lysosome inhibition significantly reduced the percentage of co-localization of LH3 and CIVC vesicles in Rab10 and Rab25, but not in double knockdown groups, suggesting that a fraction of the LH3 or Col IV pool is degraded when trafficking is disrupted (Fig. 3k). We next expressed WT, CA and DN Rab10 versions in ECs cultured in SS media. Wild-type and CA Rab10 overexpression did not affect LH3 transport to CIVC vesicles; however, the DN Rab10 mutant alone reduced LH3/CIVC vesicle co-localization by 50% (Fig. 3l, m), a finding congruent with knockdown of Rab10.

Given the hypothesis that Rab10 and Rab25 function in coordination to deliver LH3 to CIVC vesicles, we would expect to find higher co-localization between the two Rabs when stimulated for secretion. Overexpression of Rab10 WT and Rab25 WT revealed a 50% co-localization in SS media, while only about 30% of the puncta show co-localization in VEGF-supplemented media (Fig. 3n; S3a). Taking a more directed approach, we co-expressed combinations of WT, CA, and DN versions of both Rab10 and Rab25 to further investigate if their co-localization is dependent on GTP-activation. Our results demonstrate that expression of the DN Rab10 or Rab25 significantly diminished co-localization compared with any combination of WT or CA, indicating an activation dependency for associating on the same vesicle population (Fig. 3o; S3b). Taken together, these results suggest that a Rab10 and Rab25 cascade is utilized to traffic LH3 to CIVC vesicles (Fig. 3p).

Given we observed extracellular secretion was reduced with loss of Rab10 or Rab25, we reasoned that CIVC vesicles, the presumed exocytic vehicle for Col IV, should be reduced as well. Knockdown of Rab10, Rab25 or LH3 (as a negative control) showed a significant reduction in the number of ECs with detectable CIVC vesicles, indicating the loss of Rab10 or Rab25 affects the formation and/or life-time of these structures (Figure S3c, d). Previous reports determined that vacuolar protein sorting (VPS) protein VPS33B was necessary for delivery of LH3 to CIVC vesicles through direct binding of Rab10 and Rab25 [30]. Knockdown of VPS33b significantly reduced Col IV secretion and LH3 trafficking to CIVC vesicles (Figure S3e–h), consistent with a requirement for Rab10 and Rab25 in trafficking LH3 to CIVC vesicles. Overall, this data suggests that Rab10 and Rab25 work cooperatively to transport LH3 to CIVC vesicles during Col IV secretion.

### Notch signaling regulates LH3 trafficking

**Fig. 4:**
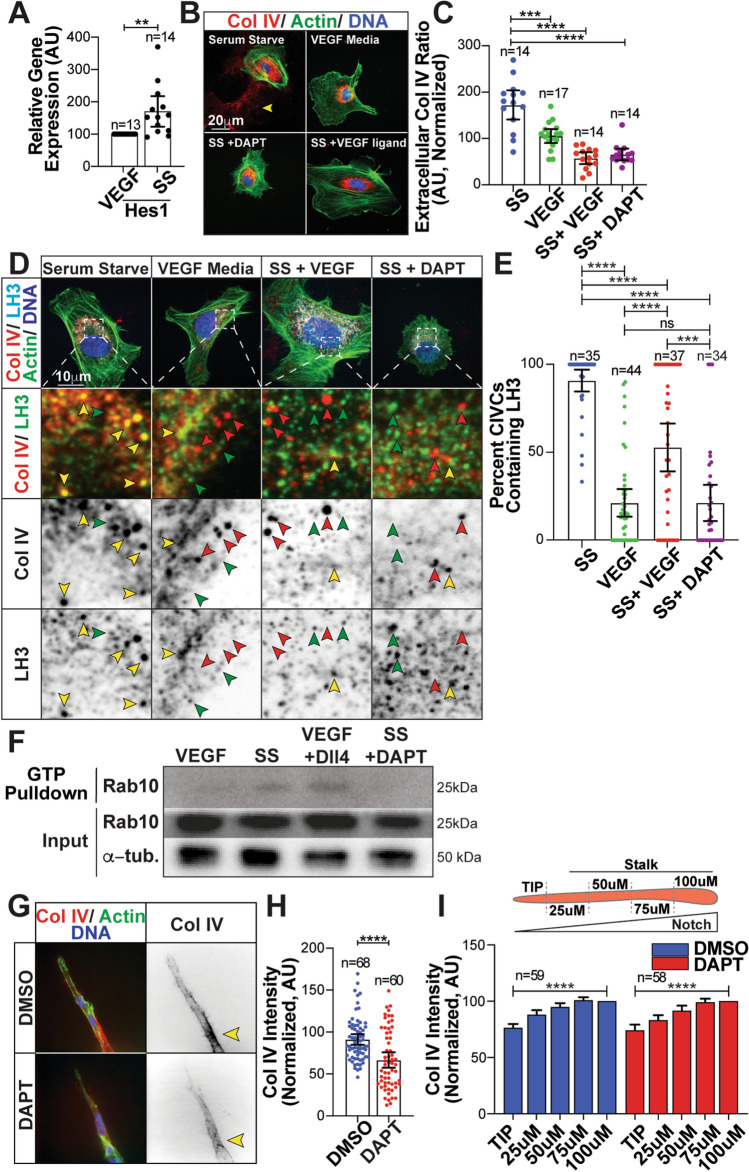
Notch signaling regulates LH3 trafficking. (**a**) Graph of relative *hes1* gene expression in ECs cultured in VEGF-containing or serum-starve (SS) media. Gene expression levels normalized to GAPDH. *N* number of replicates. (**b**) Representative images of ECs cultured in VEGF-containing, SS media, SS media + VEGF ligand (100 ng/µL), or SS media + DAPT (10 µmol/L) and stained for Collagen IV (Col IV) (red), actin (green), and DNA (blue). Arrowhead denotes extracellular Col IV secretion. (**c**) Graph of Col IV extracellular ratio of ECs across indicated groups. (**d**) Representative images of ECs cultured in indicated groups and stained for Col IV (red), LH3 (light blue), actin (green), and DNA (blue). Yellow arrowheads indicate co-localized puncta only, red arrowheads indicate Col IV only puncta, and green arrowheads indicate LH3 puncta only. (**e**) Graph of percent CIVC vesicles co-localized with LH3 in indicated conditions. (**f**) Immunoprecipitation blot for active (GTP-bound) Rab10 in the presence of VEGF-supplemented or SS media, VEGF media on a Dll4-coated plate, and SS media containing DAPT. (**g**) Representative image of fibrin-bead sprout stained for Col IV (red), actin (green) and DNA (blue). Arrowheads denote Col IV accumulation. (**h**) Graph of Col IV intensity between indicated groups. *N* number of sprouts. (**i**) Top- schematic of Col IV measurements taken on sprouts. Graph of Col IV intensity starting at the vascular front and measured back every 50 µm between indicated groups. Intensities are normalized to Col IV levels at 100 μm for each group. For all graphs *n* number of cells unless otherwise indicated. For all experiments, data represented as mean ± 95% confidence intervals. Black bars indicate comparison groups with indicated *p*-values. All *p*-values are from two-tailed Student’s *t*-test from at least three experiments. *p ≤ 0.05; **p ≤ 0.01; ***p ≤ 0.001; ****p ≤ 0.0001; *ns* not significant

Notch signaling in vascular development has been shown to control gene transcription networks critical for blood vessel maturation [19, 31, 32]. Given Col IV BM secretion is associated with more stable, quiescent blood vessels, we sought to determine if Notch signaling intersected with LH3 trafficking via Rab10 and Rab25 to control Col IV secretion. We previously determined that VEGF supplementation largely inhibited Col IV secretion, while SS media greatly increased Col IV secretion (Fig. 2h, i). In each condition, we assayed for the Notch transcriptional target Hes1 and found that in the SS condition this transcript was significantly elevated, reflecting high-Notch activation (Fig. 4a; S4a). This increase in Hes1 mRNA was also confirmed by quantifying nuclear accumulation of Hes1 protein between groups (Figure S4b, c). Next, we compared Col IV secretion in the elevated Notch SS condition to SS media supplemented with either VEGF, or Notch inhibitor DAPT. Serum-starved ECs significantly increased Col IV secretion as shown previously; however, both VEGF or DAPT administration significantly reduced Col IV secretion (Fig. 4b, c; S4d, e). To determine if Notch activity was influencing LH3 trafficking we measured co-localization of LH3 and CIVC vesicles with and without DAPT. Across all conditions, Col IV puncta were present indicating that Col IV transcription was not changed despite Notch inhibition. Notch inactivation dramatically reduced LH3/CIVC vesicle co-localization to an even greater extent than VEGF treatment, in reference to a SS control (Fig. 4d, e). Given Rab10 GTPase activity dictates LH3 trafficking to CIVC vesicles we wanted to determine if Notch was influencing Rab10 GTPase activity. To do so we probed for active (GTP-bound) Rab10 and found that SS media or plating cells on Dll4 ligand even in the presence of VEGF increased GTP-Rab10, while VEGF or DAPT greatly diminished GTP-Rab10 levels (Fig. 4f). Overall, these data suggest that Notch signaling is required for LH3 transport to CIVC vesicles through activation of Rab10.

To determine if Notch activity was related to Col IV deposition during sprouting, we treated fibrin-bead generated sprouts with DAPT. DAPT-mediated Notch inhibition increase filopodia formation consistent with other reports (Figure S4f, g) [27]. In line with our single cell analysis, DAPT treatment significantly reduced sprout Col IV levels compared with controls (Fig. 4g, h). The general consensus in the literature is that Notch activation is low in the tip cell(s) and becomes more activated in the trailing stalk cells [24, 33–38]. Thus, if Notch activation is regulating Col IV trafficking and secretion to some extent then the tip-stalk Notch gradient may be reflected in Col IV secretion. To test this, we measured Col IV intensity in 25 µm increments starting at the sprout tip moving down the stalk with and without DAPT. Consistent with low-Notch activation in the tip cell and high-Notch in the stalk cells, we observed that the Col IV intensity was the lowest at the tip region and highest in the stalk (Fig. 4i). Although whole sprout Col IV levels were reduced in DAPT-treated sprouts, this gradient (e.g., low Col IV at tip and high Col IV in stalk) was preserved in the absence of Notch activation. These data indicate that Notch activation influences Col IV secretion in sprouts.

### Notch signaling regulates Rab10 GTPase activity through DENNd4C

**Fig. 5:**
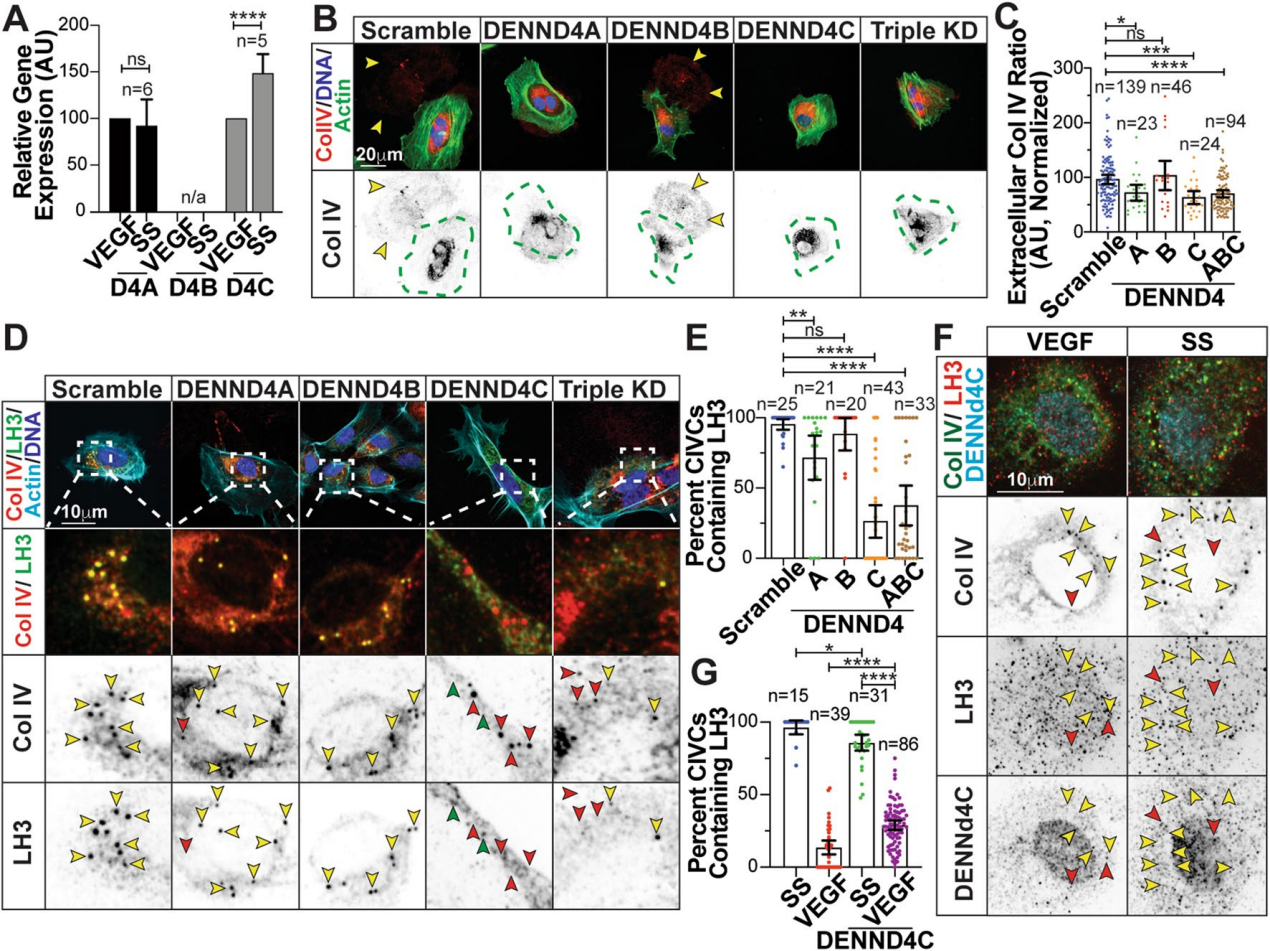
Notch signaling regulates Rab10 GTPase activity though DENNd4C. (**a**) Graph of relative gene expression of DENNd4A,B,C in ECs cultured in VEGF-containing or serum-starve (SS) media. Gene expression levels normalized to GAPDH. *N* number of replicates. (**b**) Representative images of indicated siRNA-treated ECs stained for collagen IV (Col IV) (red), actin (green), and DNA (blue). Dotted green lines indicates cell outline. Arrowheads denote extracellular Col IV secretion. (**c**) Graph of collagen IV (Col IV) extracellular ratio in indicated siRNA-treated ECs. (**d**) Representative images of indicated siRNA-treated ECs stained for Col IV (red), LH3 (green), actin (light blue), and DNA (blue). Yellow arrowheads indicate co-localized puncta only, red arrowheads indicate Col IV only puncta, and green arrowheads indicate LH3 puncta only. (**e**) Graph of percent CIVCs containing LH3 in indicated siRNA-treated ECs. (**f**) Representative images of ECs expressing DENNd4C-flag and stained for Col IV (green) and LH3 (red). Yellow arrowheads indicate Col IV-containing vesicles co-localized with LH3 and red arrowheads indicate LH3 co-localized with DENNd4C-flag. (**g**) Graph of percent Col IV-containing vesicles with LH3 in ECs expressing DENNd4C-flag in indicated culture media. For all graphs *n* number of cells unless otherwise indicated. Black bars indicate comparison groups with indicated *p*-values. All *p*-values are from two-tailed Student’s *t*-test from at least three experiments. *p ≤ 0.05; **p ≤ 0.01; ***p ≤ 0.001; ****p ≤ 0.0001; *ns* not significant

Rabs operate in a cascade mechanism where guanine exchange factors (GEFs) convert Rabs from GDP- ‘off’ to GTP-bound ‘on’ states [39, 40]. DENNd4A,B, and C have been implicated in the activation of Rab10 [5, 41]. Interestingly, Rab25 is an atypical Rab that does not have an identified GEF and likely functions more akin to a Rab effector [42, 43]. Our data suggests that activation of Rab10 is required for LH3 transport to CIVC vesicles, thus we explored the idea that Notch activity governs Rab10 activity through differential GEF expression. To test this we performed expression analysis between the Notch-low and Notch-high media conditions and observed that only DENNd4C was significantly upregulated in the SS state compared with the other DENNd4s (Fig. 5a), suggesting that Notch activation can directly modulate this transcript. Knockdown of both DENNd4A and DENNd4C, but not DENNd4B, reduced Col IV secretion compared with controls (Fig. 5b, c). However, DENNd4C produced the most significantly reduction in LH3 co-localization with CIVC vesicles (Fig. 5d, e). Next, we overexpressed DENNd4C in the presence or absence of VEGF. Overexpression of DENNd4C localized with Rab10 and partially rescued LH3 localization to CIVC vesicles in the presence of VEGF (Fig. 5f, g; S5a). These results indicated that DENNd4C is an indirect Notch target. Additionally, this provides further evidence that Rab10 activation by DENNd4C is required for LH3 trafficking and Col IV secretion.

**Fig. 6:**
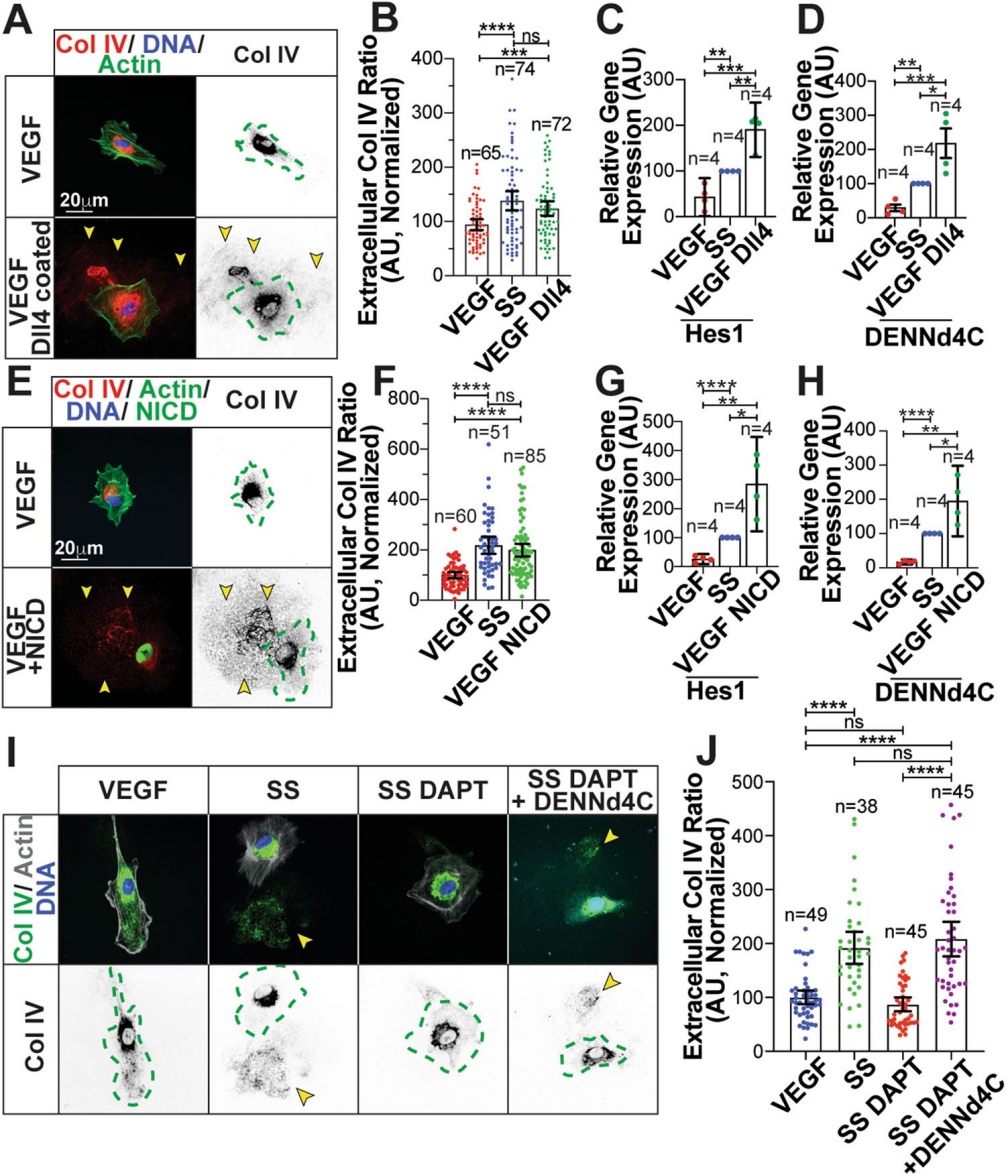
Dll4 and Notch-intracellular domain alters collagen IV secretion and gene expression. (**a**) Representative images of ECs plated on coverslips coated with recombinant protein delta like ligand 4 (Dll4) (1ug/ml). (**b**) Graph of collagen IV (Col IV) extracellular ratio in ECs plated on Dll4 coated coverslips compared to culture media controls. *N* number of cells. (**c**) Graph of relative Hes1 gene expression in ECs cultured in VEGF-containing, serum-starve (SS) media or plated on Dll4 ligand.* N* number of replicates. (**d**) Graph of relative DENNd4C gene expression in ECs cultured in either VEGF-containing, SS media or plated on Dll4. *N* number of replicates. (**e**) Representative images of ECs expressing Notch1 intracellular domain (NICD) cultured in VEGF-containing media. *N *number of replicates. (**f**) Graph of Col IV extracellular ratio in ECs between indicated groups. *N* number of cells. (**g**) Graph of relative Hes1 gene expression in ECs between indicated groups. *N* number of replicates. (**h**) Graph of relative DENNd4C gene expression in ECs between indicated groups. *N* number of replicates. (**i**) Representative images of ECs stained for Col IV (green), actin (grey), DNA (blue) and flag (light blue) cultured with VEGF, in SS media, in SS media with DAPT, or in SS media with DAPT overexpressing DENNd4C-flag. (**j**) Graph of Col IV extracellular ratio in indicated groups. *N* number of cells. For all experiments, data represented as mean ± 95% confidence intervals. Black bars indicate comparison groups with indicated *p*-values. All *p*-values are from two-tailed Student’s *t*-test from duplicate experiments. *p ≤ 0.05; **p ≤ 0.01; ***p ≤ 0.001; ****p ≤ 0.0001; *ns* not significant

Next, we wanted to further confirm the link between Notch activation and DENNd4C expression. To do so, we first plated ECs on Dll4-coated plates to promote canonical Notch activation through ligand/receptor engagement. ECs plated on Dll4 ligand in the presence of VEGF demonstrated significantly elevated Col IV secretion (Fig. 6a, b) as well as Hes1 and DENNd4C mRNA expression in reference to a VEGF-treated control (Fig. 6c, d). In a similar approach, we transduced ECs with a Notch intracellular domain (NICD) overexpressing virus to promote Notch activation. Overexpression of the NICD significantly increased Col IV secretion as well Hes1 and DENNd4C expression even in the presence of VEGF (Fig. 6e–h). Lastly, we overexpressed DENNd4C with DAPT to determine if this could rescue the Notch-mediated reduction in Col IV secretion. Overexpression of DENNd4C in the presence of DAPT rescued Col IV secretion levels to that of SS controls (Figure S6i, j). These data strongly implicate Notch signaling as an upstream modulator of the Rab10 GEF DENNd4C as well as the necessity for Rab10 activation for Col IV secretion.

### Rab10 influences Col IV bioavailability in vivo

To explore if Rab10 was required in vivo we turned to a mouse model of blood vessel development. Homozygous loss of Rab10 was lethal at embryonic day 7, consistent with other reports [44]. Rab10 heterozygous mice (Rab10^em1(IMPC)J^) were viable and did not show any appreciable differences in survival compared with WT littermates. To examine if Rab10 heterozygosity impacted Col IV bioavailability and, subsequently, sprouting angiogenesis we examined the retinal vascular plexus [27]. There was no difference in sprouting parameters between Rab10^+/−^ and WT littermates at postnatal (P) day 6 (Figure S6a–c). This was somewhat surprising as these mice have been shown to have eye development issues such as iris synechia as reported by the *International Mouse Phenotyping Consortium* (IMPC). In the retina, there was a ~ 70% reduction in Col IV/LH3 co-localization in the Rab10^+/−^ group compared with WTs (Figure S6d, e). We explored for potential reduced Col IV abundance in other tissue beds using alternating serial sections of H&E staining and Col IV immunohistochemistry to compare the same anatomical location between groups. Brain and dermal vasculature showed a reduction in Col IV staining (Figure S6f, g). These data suggest that Rab10 haploinsufficiency is associated with reduced perivascular Col IV.

To subvert the effect of global Rab10 loss of function, we mosaically over-expressed TagRFP-tagged Rab10 WT, CA or DN in the blood vessels of 72 hpf zebrafish. The zebrafish Rab10 amino acid sequence is 97% identical to the human ortholog (Figure S6h). The resulting zebrafish showed mosaic vascular expression of Rab10 variants with no visible impact on body plan or intersomitic vessel development (Figure S6i, j). Again, staining for Col IV in the cerebral vasculature, we observed that the Rab10 DN mutant alone reduced perivascular Col IV compared with overexpression of WT and CA Rab10 constructs (Figure S6k). Strikingly, at 72 hpf fish injected with the DN Rab10 demonstrated elevated frequencies of cerebral hemorrhage and pericardial effusion, suggesting compromised blood vessel integrity (Fig. S6l, m). These results indicate that loss of Rab10 impairs Col IV bioavailability in vivo.

### Loss of endothelial Notch impairs Col IV deposition in vivo

**Fig. 7:**
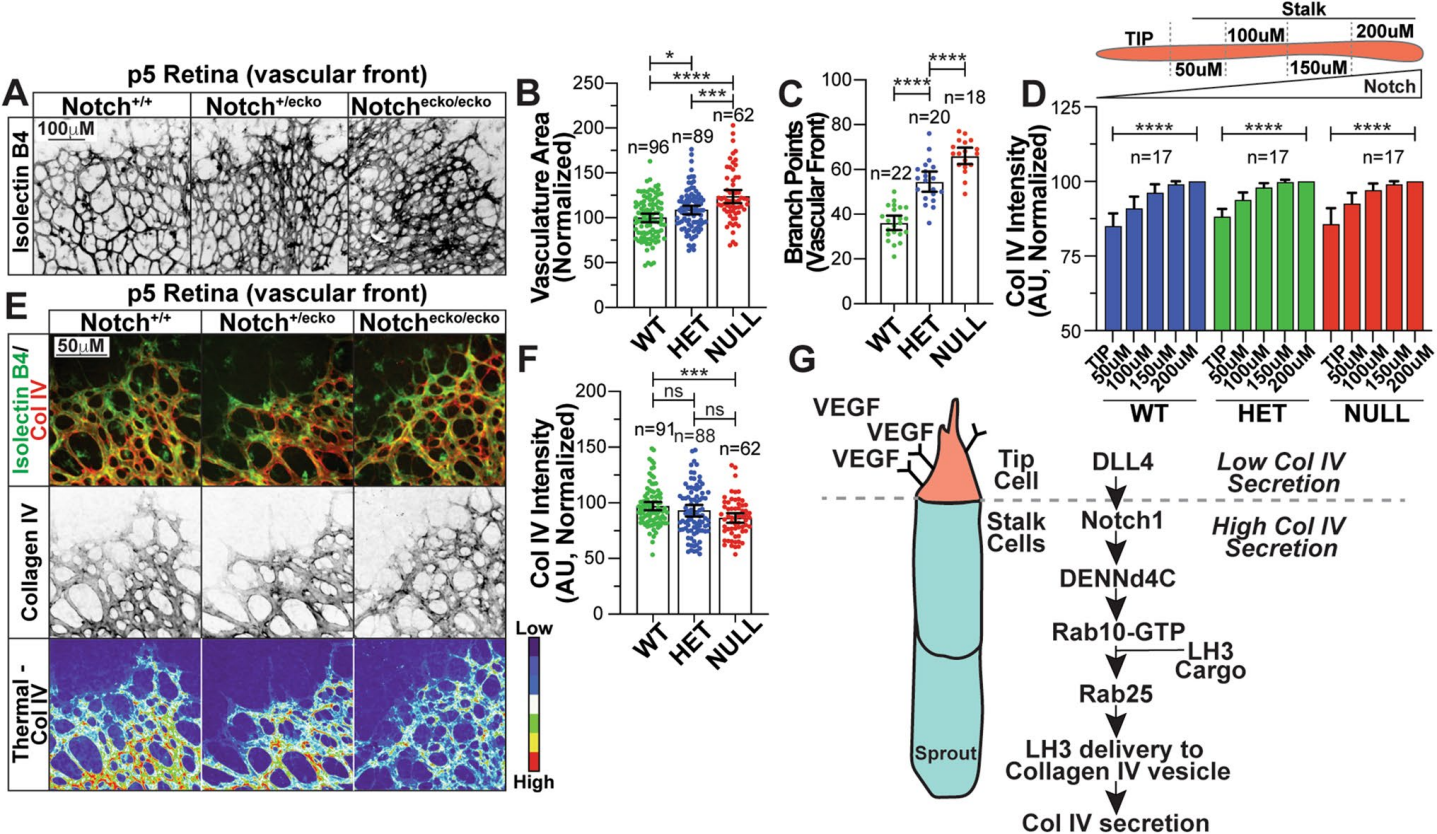
Notch1 deficient mouse retinas have reduced collagen IV. (**a**) Representative images of wild-type (WT; Cdh5-PAC-CreER^+/−^; Notch^+/+^), Notch EC knockout^(ecko)/−^ (HET, Cdh5-PAC-CreER^+/−^; Notch^flox/+^), or Notch^ecko/ecko^ (NULL, Cdh5-PAC-CreER^+/−^; Notch^flox/flox^) retinas harvested at P5 and stained for isolectin B4 (IB4). (**b**) Graph of vasculature area for vessels between indicate genotypes. (**c**) Graph of number of branch points at vascular front. (**d**) Top- schematic of Col IV measurements taken on sprouts. Graph of collagen IV (Col IV) intensity starting at the vascular front and measured back every 50 μm between indicated groups. Intensities are normalized to Col IV levels at 200 μm for each group. (**e**) Representative images of retinas harvested at P5 and stained for Col IV (red), DNA (blue), and IB4 (green) to identify blood vessels between indicated genotypes. (**f**) Graph of Col IV fluorescence intensity across indicated groups. (**g**) Model- VEGF-binding at the tip cells decreases Notch activation. In stalk cells, Notch activation promotes expression of DENNd4c which activates Rab10. Active Rab10 works with Rab25 to traffic Lysyl hydroxylase 3 (LH3) to Col IV-containing vesicles, allowing for secretion of Col IV into the extracellular space. For all experiments, data represented as mean ± 95% confidence intervals. *N* number of measurements. In all conditions, 6 or more mice were used per group. Black bars indicate comparison groups with indicated *p*-values. All *p*-values are from two-tailed Student’s t-test from duplicate experiments. *p ≤ 0.05; **p ≤ 0.01; ***p ≤ 0.001; ****p ≤ 0.0001; ns, not significant

Our in vitro results demonstrate that inhibition of Notch signaling reduces Rab10 activation and LH3 trafficking, halting Col IV secretion. To test this in vivo, we employed an endothelial-specific Notch1 knockout (ECKO) mouse model (Cdh5-PAC-CreER^+/−^; Notch1^flox/flox^) [45, 46] to explore the contribution of Notch signaling to vascular Col IV deposition. Homozygous loss of Notch1 demonstrated a stark EC overgrowth phenotype as others have reported for Notch loss of function (Fig. 7a–c) [19, 31, 47, 48]. First, we compared the relative levels of Col IV at the vascular front of P5 mouse retinas to the trailing vasculature. As mentioned, it has been previously established that ECs at the vascular front are VEGF-responsive and Notch-low, while the trailing stalk cells are more stabilized and Notch-high [36]. Col IV levels were significantly reduced at the vascular front and showed a graded increase moving into the trailing vasculature. Consistent with our fibrin-bead sprouting results, there was no trend difference of Col IV deposition between each genotype, such that, all groups showed lower Col IV at tip and higher in the stalk cells (Fig. 7d). Next, we compared the amount of perivascular Col IV levels between groups. Comparison of WT to Notch1^ecko/ecko^ showed a significant reduction in Col IV protein (Fig. 7e, f). This data are congruent with our in vitro results showing that Notch signaling is required for Col IV secretion.

## Discussion

Despite the major biological requirement of Col IV BM for blood vessel integrity and homeostasis, very little is understood about its regulation during angiogenesis. Moreover, how Col IV, or other critical BM proteins are regulated by non-transcriptional programs in ECs is largely unknown. To our knowledge, this is the first investigation posing a direct link between the regulation of Col IV secretion in angiogenesis through modulation of trafficking mediators by way of permissive Notch signaling. Our results demonstrate that Rab10 works in combination with Rab25 to transport LH3 to CIVC vesicles staged for secretion. In the absence of Rab10 or Rab25, LH3 transport is halted and Col IV secretion in ECs is dramatically attenuated. Putting this trafficking paradigm into a larger angiogenic framework, we discovered that Notch signaling is required for Rab10 activation, which seems to be the signaling bottleneck for LH3 trafficking and subsequent Col IV secretion. Overall, our data illustrate how Notch-based maturation signaling can influence trafficking mediators, providing a critical level of regulation in Col IV secretion during blood vessel development (Fig. 7g).

Col IV is highly conserved and can be traced down to the earliest bilaterians [49]. Col IV itself is expressed, to some extent, in every vertebrate tissue as an integral BM protein. It is well-established that Col IV is highly enriched in blood vessels and contributes to the overall vascular BM [50]. Col IV is not necessary for angiogenesis; however, Col IV is required for blood vessel maturation and homeostasis [15]. This enrichment is related to the ability of blood vessels to resist the mechanical strain of circulation. Mutations in Col IV (alpha 1 or alpha 2) in human patients confirm the foundational requirement of Col IV in blood vessel integrity. The primary clinical manifestation of individuals with SVD is intracerebral hemorrhage and microbleeds increasing morbidity and mortality in this affected population [16]. Rodent experimental models of Col IV mutations strongly echo results in human cohorts in demonstrating that loss of Col IV function or availability results in either embryonic lethality or early postnatal death by way of hemorrhage [15]. These observations clearly indicate that Col IV bioavailability is paramount for blood vessel homeostasis and normal life expectancy.

Our investigation in determining post-transcriptional factors that regulate Col IV secretion in ECs discovered that Rab10 and Rab25 are major trafficking mediators. Rab10 in particular has been implicated in a myriad of processes ranging from GLUT trafficking to regulating ER dynamics [51, 52]. In our hands, Rab10 echoed previous reports in *Drosophila melanogaster* egg chamber development by affecting Col IV secretion; although, our data indicated that Rab10 is an indirect mediator of Col IV trafficking [5]. Our results were congruent with a second report observing that LH3 post-Golgi sorting is controlled, in part, by Rab10 and Rab25 in mouse epithelial cells [30]. Extending these observations in primary ECs, we found that LH3 was indeed required for Col IV secretion, and LH3s delivery to CIVC vesicles was paramount to this process. Interestingly, we also found that Rab10 was the major regulatory step as compared with Rab25. Rab25 is an atypical Rab GTPase that does not possess a canonical GEF for its activation, and thus may behave more akin to a Rab10 effector. While Rab25 was necessary for LH3 trafficking and Col IV secretion, its precise role in this cascade has yet to be determined. On the other hand, Rab10 has three previously reported activating GEFs, DENNd4 A, B, and C, requiring a more classical GEF-dependent activation.

Given the stark dependence on Rab10 for LH3 trafficking to CIVC vesicles, we were very intrigued by what upstream mechanisms control Rab10 activity and how they might interface with blood vessel maturation programs. Notch signaling is fundamental to angiogenesis and adult blood vessel homeostasis [19]. In aggregate, Notch activation is repressive, decreasing EC migration and proliferation programs and is generally associated with heightened vessel maturity [19]. In the absence of Notch, blood vessels demonstrate a chronic sprouting phenotype marked by unchecked proliferation and overgrowth [31, 34]. We show in ECs that Notch activation is capable of orchestrating LH3 trafficking to CIVC vesicles by controlling transcription of the Rab10 GEF, DENNd4C. In multicellular sprouts and in mouse retina vasculature we also found that reducing Notch stabilization programs resulted in reduced overall Col IV secretion. Congruent with the notion of low-Notch in the tip region and high-Notch in the stalk cells [31, 35, 48, 53] we observed a low-to-high Col IV secretion gradient. However, both in vitro and in vivo, loss of Notch signaling did not disrupt this low Col IV at the tip and high Col IV in the stalk region. This could potentially indicate other mechanisms at play regulating Col IV secretion mechanisms other than Notch. For instance, Rasa1 has been shown to control Col IV folding and export from the ER in blood vessels [54]; it is tempting to speculate that signaling networks such as Rasa1 or the accompanying MAPK pathway could also influence post-Golgi Col IV trafficking mechanisms. Overall, we believe our findings have important implications in providing evidence that permissive Notch signaling can also interface with trafficking mediators in a comprehensive top-down regulatory response during angiogenesis.

We observed that the administration of the powerful angiocrine factor, VEGF, effectively shut down Col IV secretion by inhibiting LH3 trafficking via reduction of Rab10 and Rab25 activation. In the context of early blood vessel development, EC migration through tissue is reliant on secretion of BM degrading enzymes, such as MMP9 [55]. Energetically, it may be more advantageous to partition ECM breakdown signaling from ECM synthesis as to not mutually undermine each process. In this study, the division between Col IV secretion and intracellular degradation was controlled by LH3 trafficking and upstream activation of Notch signaling. It is well-established that in a growing sprouts, the leading tip cell has low-Notch and the trailing stalk cells have high-Notch activation [36, 48]. Our results closely adhere to this model. In a low-Notch state, the tip cell has elevated VEGFR2 expression, thus is experiencing more VEGF signaling and, according to our findings, would reduce secretion of Col IV; potentially shunting more energetic resources to migration and ECM degradation. However, in the stalk cells where Notch activation is elevated, these ECs are buttressing the newly made vascular tunnel by secreting Col IV by, in part, activated LH3 trafficking. To our knowledge, this is the first link between Notch signaling and regulation of vascular BM secretion at the post-Golgi trafficking level.

Our results bring into question how Notch may directly impact the trafficking of other critical BM proteins necessary for blood vessel integrity. For instance, others have reported that laminin-111 binds receptors and activates Dll4 signaling [56, 57]. One may speculate that the impact of Notch on the secretion machinery may elicit a feed-forward mechanism in which secretion of BM components binds integrin receptors that reinforce Notch activation. This type of cascade could explain cell–cell independent Notch signaling for sustained activation of blood vessel maturity programs required for adult vascular homeostasis. However, the organotypic trafficking programs that govern secretion of Col IV and other BM components are largely un-mapped and will require future investigations.

### Material and methods

Additional experimental procedures and a list of used materials is included in the Data Supplement. The authors will make their raw data, analytic methods, and study materials available to other researchers upon written request.

#### DNA cloning

Unless otherwise stated, all vectors were generated by PCR amplification of the desired middle element using attL1/L2- flanked oligonucleotide primers, followed by an LR reaction with either pLenti_705 (17392, Addgene) or pLEX_307 (41392, Addgene). A Gibson assembly reaction was performed with the desired gene to be assembled into an EcoRI-BamHI linearized pME-MCS destination vector. Rab10 clones were a gift from Dr. Mark McNiven (Mayo Center for Biomedical Discovery). Full-length human Rab25 cDNA was purchased from Origene (RC203413, ORIgene) and cloned into pME-MCS as described supplementary table 1. Point mutations were introduced via a Q5 site-directed mutagenesis kit (E0554S, NEB) using primers described in supplementary table 1. For co-expression of Rab10 and Rab25 in HUVECs, the destination plasmid pShuttle-CMV (16403, Addgene) was used. To create relative similar levels of expression the two genes of interest were fused together via a p2a viral DNA element. The primers used to clone tRFP-Rab10 and BFP-Rab25 are shown in supplementary table 1. A Gibson assembly was used to assemble all desired elements into the XhoI, EcoRV linearized pShuttle-CMV. All constructs were verified by sequencing.

#### Cell culture

Primary human umbilical vein cells (HUVECs; PromoCell) were cultured in EBM-2 medium supplemented with 5% fetal bovine serum (FBA), 1% penicillin/streptomycin and 1% growth supplemental kit (EGM-2). Only cells in passages 2–10 were used for experiments. Human aortic endothelial cells (HAECs) (ACBRI375, Cell-Systems), human brain microvasculature endothelial cells (HBMECs) (ACBRI376, Cell-Systems) and human dermal microvasculature endothelial cells (HDMECs) (CSC2M1, Cell-Systems) were all cultured in EGM-2. The serum-starve medium is composed of Optimem (11058021, ThermoSci) supplemented with 1% FBS (25-514, GeneseeSci) and 1% penicillin/streptomycin (P4333, Sigma). Human lung fibroblasts (NHLFs) (CC-2512, Lonza) were cultured in Dulbecco’s modified Eagle’s medium (DMEM) (25-501B, GeneseeSci) media supplemented with 10% FBS and 1% penicillin/streptomycin. Human embryonic kidney cells (HEKs) (85120602, Sigma) were cultured in DMEM media supplemented with 10% FBS and 1% penicillin/streptomycin. All cells were grown at 37 °C in a humidified atmosphere with 5% CO_2_. Adenovirus strain adNICD-GFP was a kind gift from Dr. Mark Sussman (San Diego State University) [58]. Brefeldin A (00-498093, ThermoSci), chloroquine (C6628, Sigma), cycloheximide (C7698, Sigma), DAPT (D5942, Sigma) and VEGF (V7259, Sigma) were used for indicated experiments. Recombinant Human Dll4 (1506-D4-050, R&D) protein was used at 1 µg/mL for coating coverslips where indicated.

#### Animal studies

Zebrafish (*Danio rerio*) were bred and housed in standard conditions in accordance with the University of Denver. The *Tg(kdrl:eGFP)* (kind gift Victoria Bautch)*, Tg(cdh5:gal4FF)* (kind gift Arndt Siekmann) were injected with *Tg(5xUAS:tRFP-Rab10)* (this study), *Tg(5xUAS:tRFP Rab10*^*Q68L*^*)* (this study), *or Tg(5xUAS:tRFP-Rab10*^*T23N*^*)* (this study) constructs with transposase at the single cell stage [59]*.* Procedures used in the experiments were approved by the Institutional Animal Care and Use Committee. For cryosectioning, zebrafish were fixed in 4% PFA overnight and dehydrated in 100% methanol for 48 h. Thereafter, embryos were briefly rehydrated in TBST and then incubated in a 30% sucrose solution for 24 h. Fish were embedded in OCT prior to sectioning and staining as previously reported [60]. Images were obtained using a Leica m165 FC Stereoscope.

Mice were bred and housed in standardized conditions in the Mouse Research Animal Facility at University of Denver and monitored regularly to maintain a pathogen-free environment. Procedures used in the experiments were approved by the Institutional Animal Care and Use Committee. Rab10^em1(IMPC)J^ mice were obtained from The Jackson Laboratory (MMRRC# 42330). Rab10^em1(IMPC)J^ pups were obtained via intercrossing of heterozygous mutants or via outcrossing with BL6 background mice. None of the intercrossed heterozygote mutant offspring were found to be homozygous null, consistent with other reports [44]. At the time of sacrifice genotypes were determined via tail-clips and PCR. Postnatal 5 Cdh5-PAC-CreER [46]; Notch1^flox/flox^ [45] mouse retinas were kindly provided by Dr. Jan Kitajewski (University of Illinois).

#### Statistics

All statistical analyses were conducted using GraphPad PRISM software. Student’s *t*-test were used to compare the difference between the control and treated group in our studies. A two-tailed P < 0.05 was significant, and the data are presented as mean $\pm$ 95% confidence interval.

### Supplemental methods

#### Collagen IV extracellular secretion and co-localization assays

Extracellular Col IV ratio was quantified by taking the total fluorescence intensity of Col IV and the fluorescence intensity inside the cell perimeter. Col IV extracellular ratio = [(total fluorescence − inside fluorescence)/inside fluorescence] × 100. A value at 1 or below is representative of little/ no Col IV secretion while a value above 1 is representative of substantial Col IV secretion. Values are then normalized to control condition values. To quantify vesicle co-localization, images with multiple channels were imported into FIJI. Then individual puncta were assigned a number using the ‘point tool’ on each channel. Only puncta that had more than 1 assigned number were scored as co-localized.

#### Endothelial cell transfection

Cells were transfected using the Neon Transfection System (MPK5000, ThermoSci) according to manufacturer’s protocol. Briefly, cells were trypsinized and washed with DPBS then suspended in a solution of R-buffer (100 µl; Invitrogen) containing either (100 µM) siRNA or (1 µg) over-expression plasmids (pLenti_705, pLEX_307 or pShuttle-CMV) using the recommended electroporation protocol (1350 V, 30 ms, 1 pulse). Thereafter, cells were either plated onto pre-treated poly-L-lysine coated glass coverslips for immunohistochemistry (IHC) and live-imaging or plated into petri dishes. IHC and live-imaging experiments were conducted 18–30 h after transfection while cell lysates were harvested 48–72 h after transfections.

#### Collagen IV-containing vesicle identification

CIVC vesicles are defined and were counted as previously reported [30]. Briefly, bright fluorescence puncta ranging from 200 to 900 nm (under 1μM) in the cytoplasm away from any ER or Golgi structures were counted as a CIVC vesicle.

#### Immunohistochemistry

Standard procedures were used for IHC[2]. Briefly, HUVECs grown on poly-L-lysine coated coverslips were washed and subsequently fixed with 4% PFA for 10 min. Cells were then washed and incubated at RT with 0.1% Triton-X for 10 min. Blocking was performed with 2% BSA prior to primary antibody incubation. Commercial antibodies used were: goat anti-Col IV (ab769, Sigma), rabbit anti-Col IV (ab6586, Abcam), rabbit anti-Laminin (L9393, Sigma), mouse anti-heparan sulfate proteoglycan (MABT12, Sigma) and mouse anti-PLOD3 (SAB1400329, Sigma). AlexaFluor conjugated secondary antibodies include donkey anti-goat 555(A32816, ThermoSci) and donkey anti-mouse 647 (A31571, ThermoSci). Hoechst 33342 (H3570, ThermoSci) used as a DNA stain. For wound healing assays, scratches were made when HUVECs were 90% confluent. Dishes were washed twice and then replaced fresh EGM-2 medium for up to 8 h before fixation with 4% PFA. CellEvent Caspase-3/7 (C10723, ThermoSci) was used to investigate transfection efficiency. All histology was performed at HistoTox Labs (Boulder, CO).

#### Sprouting assay

A fibrin-bead sprouting assay was conducted as described by Nakatsu et al. [23]. Briefly, after trypsinization, HUVECs were incubated with cytodex3 microcarrier beads (C3275, Sigma) at a ratio of 400 cells per bead. The samples were incubated for 4 h with agitation every 15 min. The mixture was then transferred to a 6cm^2^ dish and cultured at 37 °C overnight. The next day, beads coated with HUVECs were collected and resuspended in a 2 mg/ml fibrinogen solution (F8630, Sigma), which contained 0.15 U/ml aprotinin (A1153, Sigma). As the beads were added to poly-L-lysine pre-treated glass coverslips, 0.625 U/ml thrombin (T4648, Sigma) was added, gently mixed, and incubated at 37 °C until the gel solidified. Then, 25,000 NHLFs which were resuspended in 1 ml EGM-2 were added on top of the gel. The media were changed every 2 days and fixed 5–8 days after embedding with 4% PFA. Standard IHC staining solutions were used. Images were obtained on inverted Nikon Ti-E spinning disk confocal and analyzed with FIJI software.

#### Protein and RNA isolation from endothelial cells

Western blotting was performed using standard procedures. Whole cell lysates were harvested for protein extraction 48–72 h after transfection. An equal amount (20–35 g) of protein was electrophoresed on 12 and 7% polyacrylamide gels and then transferred to nitrocellulose membranes. The membrane was blocked in ~ 5% milk or 2% BSA followed by antibody incubation overnight at 4C. Antibodies used are listed below: rabbit anti- α-tubulin (ab52866, Abcam); mouse anti-Rab10 (MABN730, Sigma); rabbit anti-Col IV (ab6586, Abcam). The internal loading control for all experiments was α-tubulin. Secondary HRPs (GeneseeSci) and ProSignal ECL substrate (20-300B, GeneseeSci) were used. For GTP associations, ECs were incubated with indicated media then lysed and incubated with guanosine 5′-triphosphate agarose beads (G9768, Sigma). RNA extraction was performed using TRIzol (15596026, ThermoSci) with standard procedures. RT-PCR was performed on cDNA libraries using high-capacity cDNA reverse transcription kit (4368814, ThermoSci) according to manufacturer instructions. PCRs were performed using ProFlex PCR System (4484073, ThermoSci).

#### Retina extraction

Eyes from male and female mice were harvested at p6 and fixed in 4% PFA for 2 h at room temperature. Immediately after fixation, retinas were dissected and flattened by making curve-relieving cuts. The retinas were then fixed for an additional 30 min. Then, retinas were placed in 2% BSA blocking solution overnight at 4C. On day 2, retinas were stained for 24 h at 4C with goat anti-collagen IV (same as IHC) and rabbit anti-PLOD3 antibody (HPA001236, Sigma). On day 3, retinas were washed twice in TBST and then stained for 24 h at 4C with conjugated Isolectin B4 (IB4) and Hoechst 33342 (see IHC), donkey anti-goat 555(same as IHC) and donkey anti-mouse 647 (same as IHC). On day 4, the specimens were washed three times in TBST for 10 min and then left in TBST overnight at 4C. On day 5, the retinas were mounted on slides and imaged. For the p5 Notch retinas, eyes were fixed in 4% PFA for 2 h and then stored in DPBS for shipping. Upon receiving the eyes, dissections and staining proceeded as stated above with the addition of a 10 min 0.1 × Triton-X incubation prior to 2% BSA blocking overnight. Collagen IV intensity reported is Mean Gray Value analysis performed in FIJI software. Only collagen IV that was co-localized with IB4 was analyzed to avoid collagen sleaves.

## Supplementary Information

Below is the link to the electronic supplementary material.

Supplementary file1 (PDF 4916 kb)
