# Correction to: Notch regulates vascular collagen IV basement membrane through modulation of lysyl hydroxylase 3 trafficking

**DOI:** 10.1007/s10456-021-09801-w

**Published:** 2021-06-08

**Authors:** Stephen J. Gross, Amelia M. Webb, Alek D. Peterlin, Jessica R. Durrant, Rachel J. Judson, Qanber Raza, Jan K. Kitajewski, Erich J. Kushner

**Affiliations:** 1 Department of Biological Sciences, University of Denver, Denver, CO 80210 USA; 2 HistoTox Labs, Boulder, CO USA; 3 Department of Physiology and Biophysics, University of Illinois, Chicago, IL USA

## Correction to: Angiogenesis 10.1007/s10456-021-09791-9

In the original publication, some of the figures were missed mistakenly in the Supplementary Information. Hence, the updated Supplementary Information is provided in this correction.

The original article has been corrected.

## Supplementary Information

Below is the link to the electronic supplementary material.

Supplementary file1 (PDF 4915 kb)

