# Supplementary material for: Notch regulates vascular collagen IV basement membrane through modulation of lysyl hydroxylase 3 trafficking
Source: Angiogenesis. 2021 May 6;24(4):789–805. doi: 10.1007/s10456-021-09791-9 (PMC8487879; doi:10.1007/s10456-021-09791-9)
Supplement: Supplementary file 1 — Supplementary file1 (PDF 4916 kb) [file 10456_2021_9791_MOESM1_ESM.pdf]

Supplemental Materials: Notch Regulates Vascular Collagen IV Basement Membrane Through Modulation of Lysyl Hydroxylase 3 Trafficking

Stephen J. Gross[1]; Amelia M. Webb[1], Alek D. Peterlin[1]; Jessica R. Durrant[2], Rachel J. Judson[1], Qanber Raza[3], Jan K. Kitajewski[3], Erich J. Kushner*[1].

[1]Department of Biological Sciences, University of Denver, Denver, CO, USA; [2]HistoTox Labs, Boulder, CO, USA;[3]Department of Physiology and Biophysics, University of Illinois, Chicago, IL, USA; *Author for correspondence: Erich Kushner, Ph.D.

SUPPLEMENTAL FIGURES

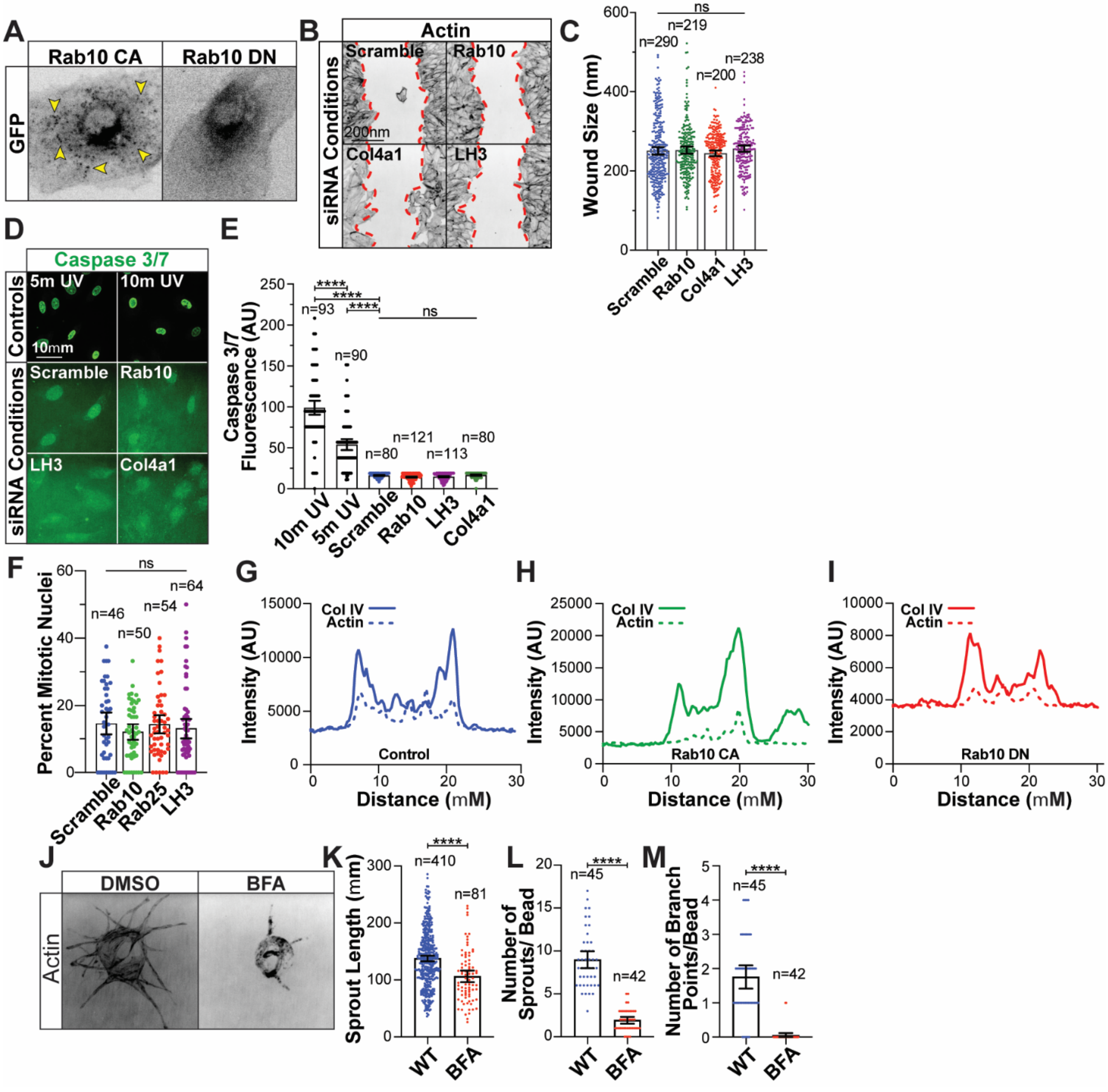

Supplemental Figure 1. Effect of Rab10 on endothelial viability markers.
(A) Representative image of constitutively active (CA) and dominant negative (DN) GFP-Rab10 localization in endothelial cells. (B) Representative images of scramble, Rab10, lysyl hydroxylase 3 (plod3), and collagen IV (Col IV, col4a1) siRNA treated ECs in scratch wound assay. Cells were stained for actin (grey) to delineate scratch wound margins. Dotted red line indicates wound border. (C) Graph of average wound size in indicated groups of indicated siRNA-treated ECs. N=number of measurements. (D) Representative images of scramble, Rab10, lysyl hydroxylase 3 (plod3), and Col IV siRNA-treated ECs stained for Caspase 3/7 activation (green). Controls were subjected to UV light exposure for indicated times to elicit caspase activation. (E) Graph of Caspase 3/7 activation fluorescence intensity in ECs treated with indicated siRNA treatment groups. Measurement of GFP fluorescence intensity within the nuclei of ECs. N=number of cells. (F) Percent mitotic nucleic as assessed by phospho-histone H3 staining between indicated siRNA treatment groups. N=number of cells. (G-I) Line scan of Col IV and actin related to figure 1L,M. (J) Representative fibrin-bead treated with either DMSO (vehicle) or brefeldin A (BFA) to inhibit secretion. (K-M) Graphs quantifying indicated sprouting parameters. N=number of sprouts. For all experiments, data represented as mean $\pm$ 95% confidence intervals. Black bars indicate comparison groups with indicated p-values. All p-values are from two-tailed Student's t-test from duplicate experiments. *$p \leq 0.05$; **$p \leq 0.01$; ***$p \leq 0.001$; ****$p \leq 0.0001$; ns, not significant.

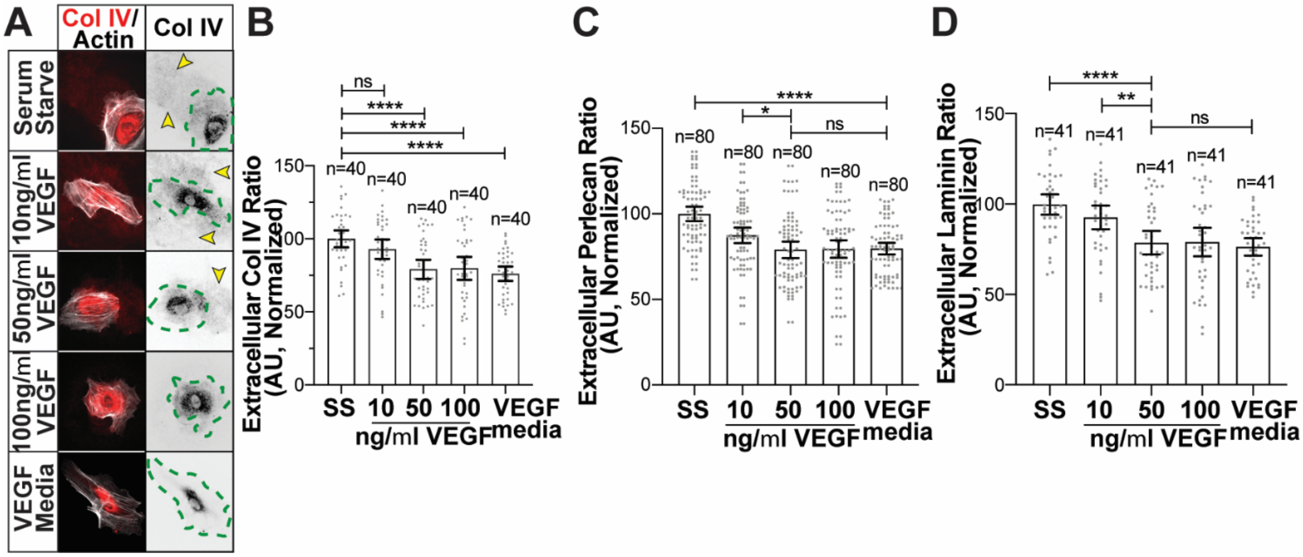

Supplemental Figure 2. Effect of VEGF on basement membrane secretion in HUVECs. (A) Representative images of ECs cultured in VEGF-containing, serum-starve (SS) media, or SS media supplemented with indicated concentrations of VEGF ligand. ECs were stained for collagen IV (Col IV) (red) and actin (grey). Dotted green line indicates cell outline. Arrowheads denote extracellular Col IV secretion. (B) Graph of extracellular Col IV ratio in ECs. (C) Graph of extracellular Perlecan ratio in ECs. (D) Graph of extracellular Laminin ratio in ECs. For all experiments, data represented as mean ± 95% confidence intervals and n=number of cells Black bars indicate comparison groups with indicated p-values. All p-values are from two-tailed Student's t-test from at least three experiments. *$p \leq 0.05$; **$p \leq 0.01$; ***$p \leq 0.001$; ****$p \leq 0.0001$; ns, not significant.

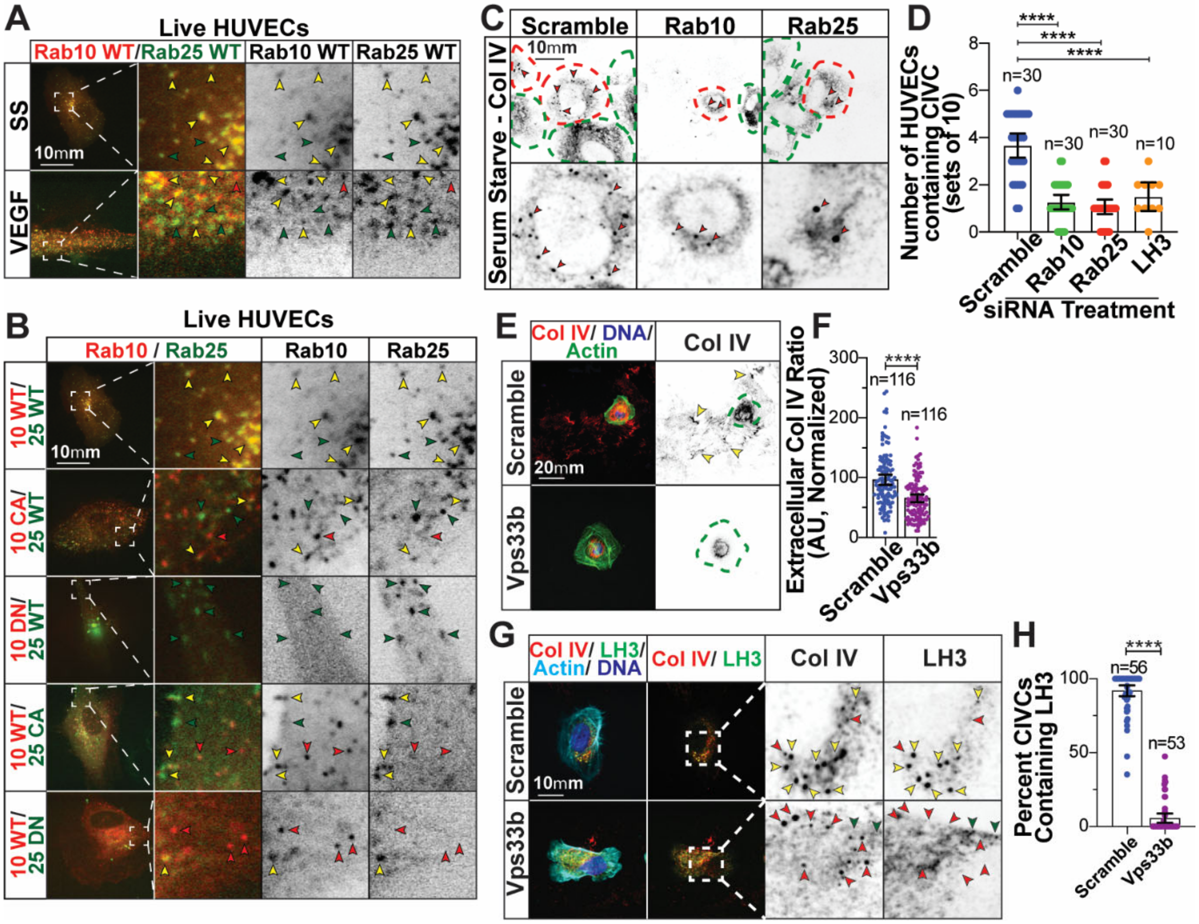

Supplemental Figure 3. Rab10 and Rab25 work in combination to traffic LH3 to CIVC vesicles.
(A) Representative images of ECs co-expressing RFP-Rab10 WT and BFP-Rab25 WT in either VEGF-containing or serum-starve (SS) media. Yellow arrowheads denote co-localized Rab10 and Rab25 puncta, red arrowheads denote Rab10 puncta only, and green arrowheads denote Rab25 puncta only. (B) Representative images of ECs transfected to co-expression RFP-Rab10 WT/CA/DN and BFP-Rab25 WT/CA/DN cultured in SS media. Yellow arrowheads denote co-localized Rab10 and Rab25 puncta, red arrowheads denote Rab10 puncta only, and green arrowheads denote Rab25 puncta only. (C) Representative images of scramble, Rab10 and Rab25 siRNA-treated ECs cultured in SS media and stained for collagen IV (Col IV) (grey). Dotted red and green lines indicates CIVC vesicle positive or negative ECs, respectively. Arrowheads denote CIVC vesicles. (D) Graph of number of ECs containing CIVC vesicles in scramble, Rab10, Rab25, and LH3 siRNA-treated ECs cultured in SS media. (E) Representative images of ECs transfected with either scramble or Vps33b siRNA and stained for Col IV (red), actin (green), and DNA (blue). Dotted green line indicates cell outline. Arrowheads denote extracellular Col IV secretion. (F) Graph of Col IV extracellular ratio of indicated siRNA treated ECs. N= number of measurements. (G) Representative images of ECs transfected with either scramble or Vps33b siRNA and stained for Col IV (red), LH3 (green), actin (light blue), and DNA (blue). Yellow arrowheads indicate co-localized puncta only, red arrowheads indicate Col IV only puncta, and green arrowheads indicate LH3 puncta only. (H) Graph of percent CIVCs containing LH3 in scrambled or Vps33b-siRNA treated ECs. For all graphs n=number of cells unless otherwise indicated. For all experiments, data represented as mean ± 95% confidence

intervals. Black bars indicate comparison groups with indicated p-values. All p-values are from two-tailed Student's t-test from at least three experiments. $*p \leq 0.05$; $**p \leq 0.01$; $***p \leq 0.001$; $****p \leq 0.0001$; ns, not significant.

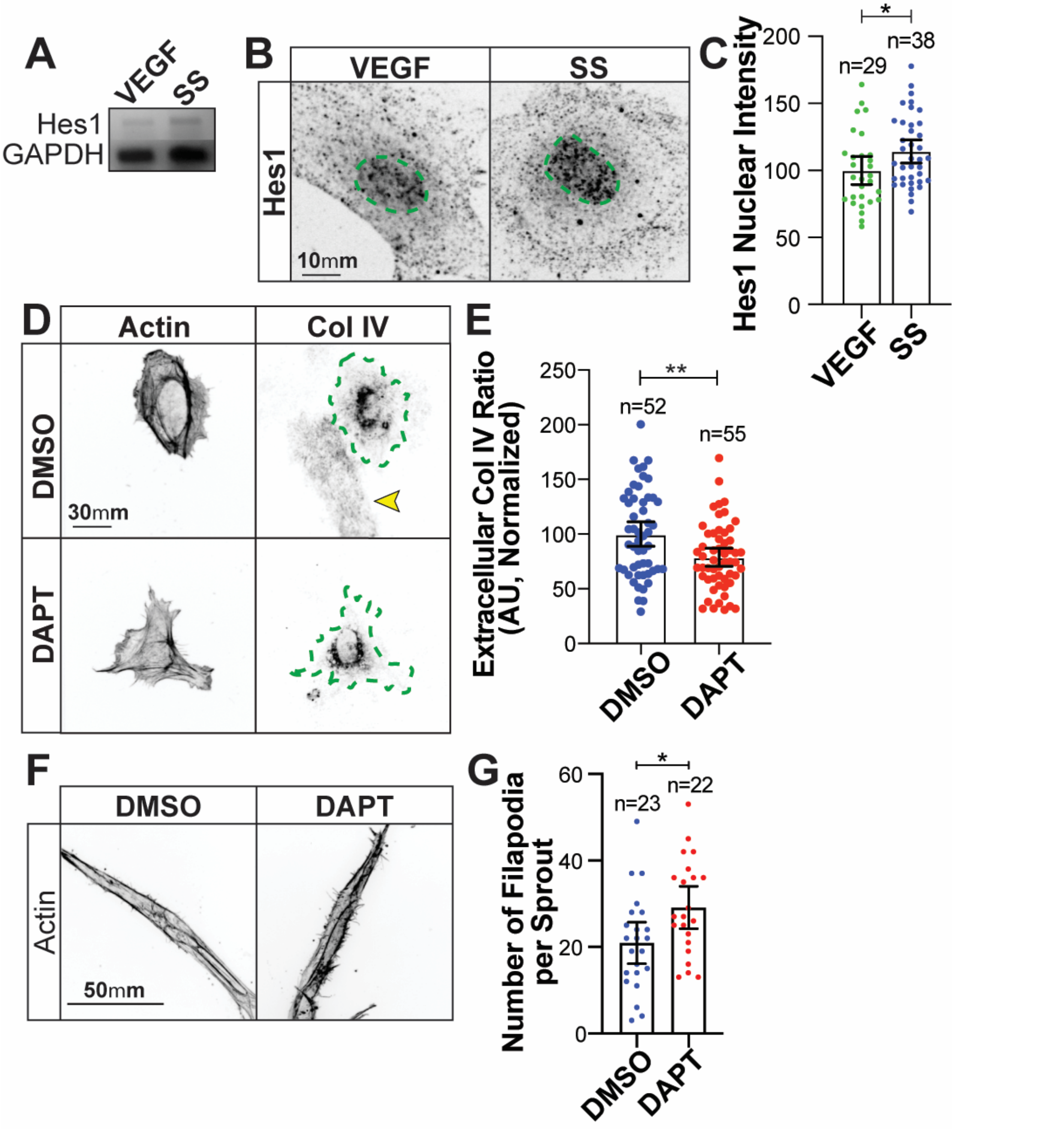

Supplemental Figure 4. Notch signaling regulated LH3 trafficking.
(A) Representative image of Hes1 gene expression ECs cultured in VEGF-containing or serum-starve (SS) media examined by RT-PCR. Gene expression levels normalized to GAPDH. (B) Representative of Hes1 staining between endothelial cells treated with VEGF or in SS media. Green line denote nucleus. (C) Graph of Hes1 intensity at nucleus between indicated groups. N=number of cells. (D) Representative images of ECs cultured in SS media with either DMSO (vehicle) or DAPT and stained for actin (left) and collagen IV (Col IV) (right). Dotted green line indicates cell outline. Arrowheads denote extracellular Col IV secretion. (E) Graph of Col IV extracellular ratio of ECs cultured in SS media with either DMSO (vehicle) or DAPT. N=number of cells. (F) Representative fibrin-bead sprout stained for actin to delineate filopodia treated with either DMSO or DAPT. (G) Graph of number of filopodia between indicated groups. N=number

of sprouts. For all experiments, data represented as mean ± 95% confidence intervals. Black bars indicate comparison groups with indicated p-values. All p-values are from two-tailed Student's t-test from at least three experiments. $^*p \leq 0.05$; $^{**}p \leq 0.01$; $^{***}p \leq 0.001$; $^{****}p \leq 0.0001$; ns, not significant.

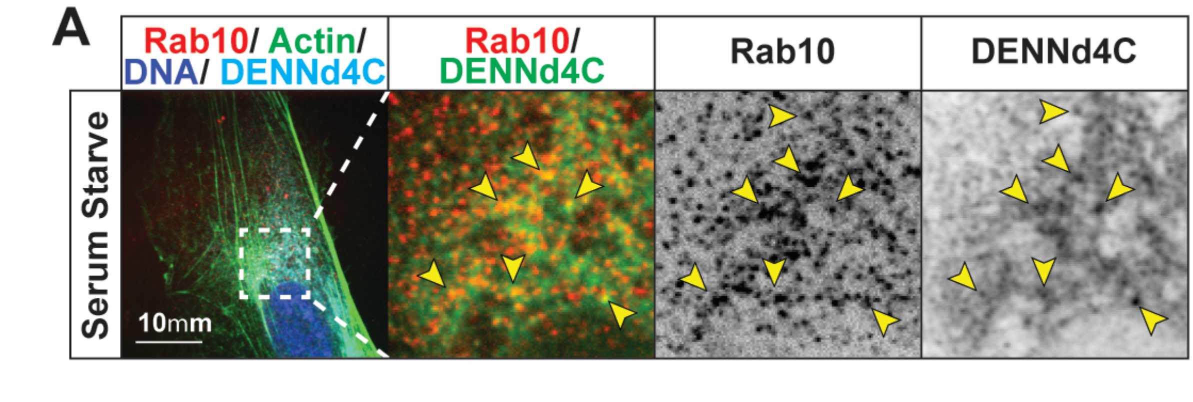

Supplemental Figure 5. Notch signaling regulates Rab10 GTPase activity through DENND4C.
(A) Representative images of ECs expressing DENND4C-flag and RFP-Rab10 WT. Yellow arrowheads indicate Rab10 puncta co-localizing with DENND4C-flag.

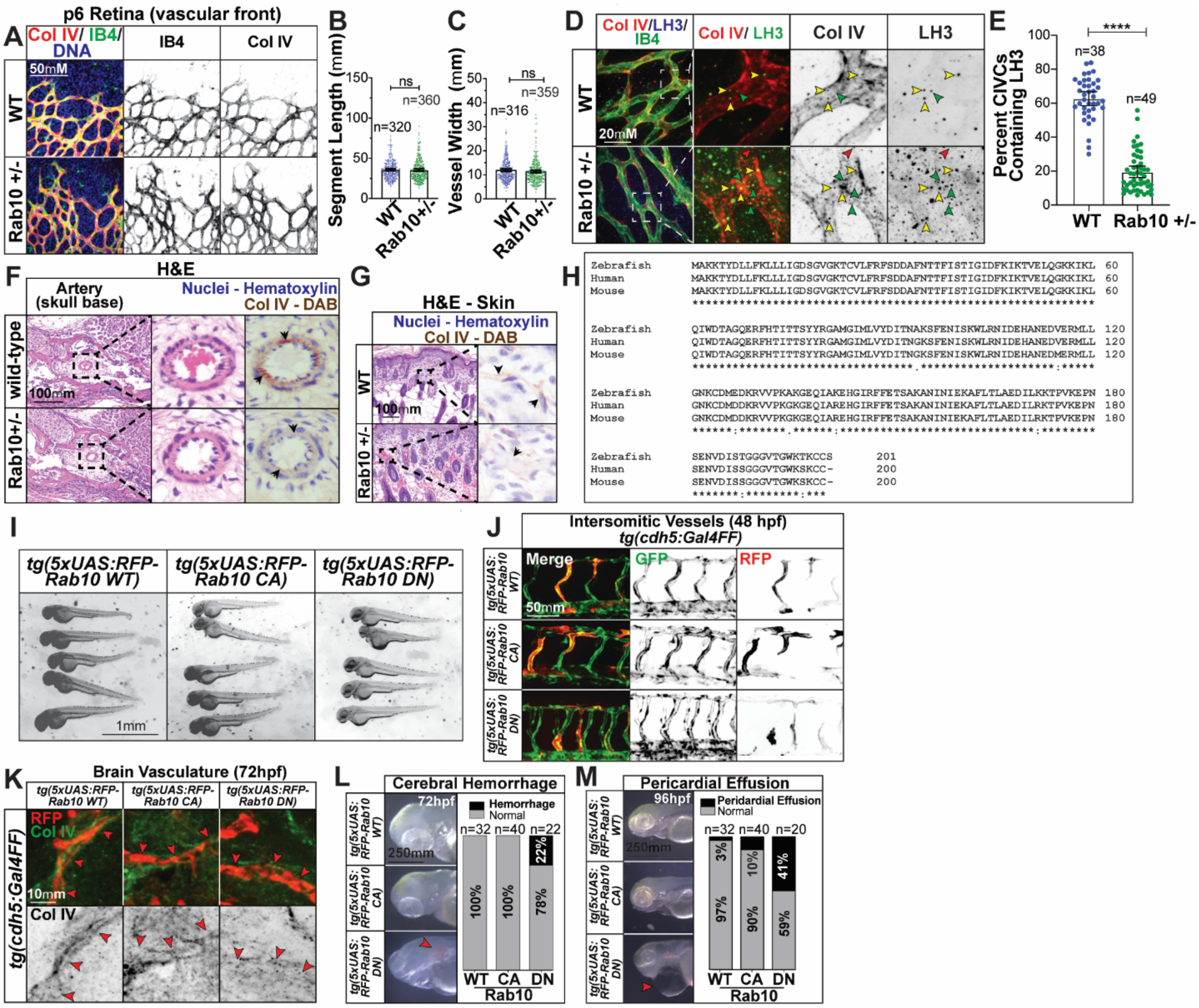

Supplemental Figure 6. Rab10 influences Collagen IV bioavailability *in vivo*.
(A) Representative images of WT or Rab10$^{+/-}$ mice retinas harvested at P6. Retinas were stained for collagen IV (Col IV) (red), DNA (blue), and conjugated-isolectin B4 (IB4) (green) to identify blood vessels. (B,C) Graph of sprouting parameters for WT or Rab10$^{+/-}$ P6 mouse retinas. N= number of measurements from at least 5 mice. (D) Representative images of WT or Rab10$^{+/-}$ mice retinas harvested at P6. Retinas were stained for Col IV (red), LH3 (blue) and IB4 (green) to identify blood vessels. (E) Graph of percent Col IV-containing vesicles with LH3 in WT or Rab10$^{+/-}$ P6 mouse retinas. N= number of measurements from at least 5 mice. (F) Hematoxylin and eosin (H&E) stained brain slices from WT or Rab10$^{+/-}$ P6 mouse retinas. Col IV stained with DAB (3-3' diaminobenzidine) indicated by arrowheads. (G) H&E stained cryosectioned skin slices from wild-type or Rab10$^{+/-}$ mice. Collagen IV stained with DAB indicated by arrowheads. (H) Amino acid alignment of Rab10a from human, zebrafish, and mouse. (I) Representative images of 48hpf expressing 5xUAS:RFP-Rab10 wild-type (WT)/ constitutively active (CA)/ dominant negative (DN). (J) Representative images of 48hpf zebrafish expressing 5xUAS:RFP-Rab10 WT/CA/DN in intersomitic blood vessels. (K) Representative images of 72hpf zebrafish brain cryosections expressing 5xUAS:RFP-Rab10 WT/CA/DN stained for Col IV. (L) Representative images of 72hpf zebrafish with 5xUAS:RFP-Rab10 WT/CA/DN injections. Arrowhead denotes cerebral hemorrhage. N= number of fish. (M) Representative images of 96hpf zebrafish with 5xUAS:RFP-Rab10 WT/CA/DN injections.

Arrowhead denotes pericardial effusions. N= number of fish. For all experiments, data represented as mean $\pm$ 95% confidence intervals. Black bars indicate comparison groups with indicated p-values. All p-values are from two-tailed Student's t-test from at least three experiments. *$p \leq 0.05$; **$p \leq 0.01$; ***$p \leq 0.001$; ****$p \leq 0.0001$; ns, not significant.

Supplemental Tables:

Gene of Interest	Forward Primer	Reverse Primer
TagRFP	gcttgatatcgttaatatggtgtctaagggcgaagag c	ggtggcgaccggtggatccgtgcttcccgaattaagtttgtgcccca gtttgctaggg
GFP	taagcttgatatcgttaatatgagtaaaggagaagaa cttttcactgga	ggcgaccggtggatccgtgcttcccgatttgtatagttcatccatgcc atgtgtaatcc
Rab10	tccaccggtcgccaccatggcgaagaagacgtac gacctg	gaactagtggatcgttttcagcagcatttgctcttccagc
BFP	gcttgatatcgttaattaagccgccaccatgagcga gctgattaaggagaaca	ccccccgccggagcccccaccgccgtgccccagtttgctaggg a
Rab25	gcggtgggggctccggcggggggggtccggg aatggaactgaggaagattataac	ctagaactagtggatcgtttaaacttagaggctgatgcaacaggccc
tRFP-Rab10 p2a	cgacgcggccgctcgaggccgccaccatggtgtc taagggcgaagagc	tgcttgctttagcagagagaagtttgtggcgccgctgccgcagcatt tgctcttccagcc
p2a BFP-Rab25	agcaagcaggtgatgttgaagaaaacccccgggcc tagcgagctgattaaggagaacatgc	ctagatccggtggatcggatatcttagaggctgatgcaacaggccc tc
Rab25 S21V - CA	ggcgaagtaggtgtggggaagac	gatcagcaccaccttgaagacaaa
Rab25 T26N - DN	gggaagaacaatctactctcccg	cacacctgattcgccgatcagc

Supplementary Table I. General Cloning Primers.
Sequences of the primer pairs used to assemble middle entry plasmids of either GFP or TagRFP versions of Rab10 WT, CA or DN as well as BFP versions of Rab25 WT, CA or DN.

Gene of Interest	Forward Primer	Reverse Primer
GAPDH	tgcaccaccaactgcttagc	ggcatggactgtggtcatgag
Col4a1 N-term	gatgaagggtgatccaggtgagatac	cttgagcttgtcctggtactcctgg
Col4a1 C-term	acagccagaccattcagatcccacc	gcacttctaaactcctccaggcagg
Hes1 A	tcaacacgaccggataaa	ccgcgagctatctttcttca
Hes1 B	tgccagctcatataatggaggaa	ccatgataggctttgatgacttt

Supplementary Table II. RT-PCR primers.
Sequences of the primers used in RT-PCR analysis of gene expression in ECs.

Common_F	ctgtttttcctttcagctcagt
WT_R	cagcatcacaggaaccaaac
Rab10_R	catttggagaaaagcatcagg

Supplementary Table III. Mouse Genotyping Primers.
Sequences of the primers used to determine the genotype of Rab10.
